# Impella Versus VA-ECMO for Patients with Cardiogenic Shock: Comprehensive Systematic Literature Review and Meta-Analyses

**DOI:** 10.3390/jcdd10040158

**Published:** 2023-04-05

**Authors:** Vittoria Ardito, Lilit Sarucanian, Carla Rognoni, Marina Pieri, Anna Mara Scandroglio, Rosanna Tarricone

**Affiliations:** 1 Centre for Research on Health and Social Care Management (CERGAS), SDA Bocconi School of Management, 20136 Milan, Italy; 2 Department of Anesthesia and Intensive Care, IRCCS San Raffaele Scientific Institute, 20132 Milan, Italy; 3 Department of Social and Political Science, Bocconi University, 20136 Milan, Italy

**Keywords:** Impella, VA-ECMO, cardiogenic shock, literature review, meta-analyses

## Abstract

Impella and VA-ECMO are two possible therapeutic courses for the treatment of patients with cardiogenic shock (CS). The study aims to perform a systematic literature review and meta-analyses of a comprehensive set of clinical and socio-economic outcomes observed when using Impella or VA-ECMO with patients under CS. A systematic literature review was performed in Medline, and Web of Science databases on 21 February 2022. Nonoverlapping studies with adult patients supported for CS with Impella or VA-ECMO were searched. Study designs including RCTs, observational studies, and economic evaluations were considered. Data on patient characteristics, type of support, and outcomes were extracted. Additionally, meta-analyses were performed on the most relevant and recurring outcomes, and results shown using forest plots. A total of 102 studies were included, 57% on Impella, 43% on VA-ECMO. The most common outcomes investigated were mortality/survival, duration of support, and bleeding. Ischemic stroke was lower in patients treated with Impella compared to the VA-ECMO population, with statistically significant difference. Socio-economic outcomes including quality of life or resource use were not reported in any study. The study highlighted areas where further data collection is needed to clarify the value of complex, new technologies in the treatment of CS that will enable comparative assessments focusing both on the health impact on patient outcomes and on the financial burden for government budgets. Future studies need to fill the gap to comply with recent regulatory updates at the European and national levels.

## 1. Introduction

Cardiogenic shock (CS) is a low cardiac output (CO) syndrome causing tissue hypoxia and eventually end-organ failure. The most frequent etiology of CS is acute myocardial infarction complicated by left ventricular systolic dysfunction. The incidence of CS in patients with ST-segment elevation myocardial infarction (STEMI) is approximately 8% [1]. Despite improvements in clinical management over time, CS is still characterized by high levels of mortality, with 30-day mortality rates around 40–60% [2,3]. 

Mechanical circulatory support (MCS) has gained a crucial role in the treatment of CS, since it is able to provide perfusion, restore cardiac output, and eventually ventricular unloading [3]. Currently, the most common weanable mechanical circulatory support (MCS) devices used in clinical practice are the intra-aortic balloon pump (IABP), the veno-arterial extra-corporeal membrane oxygenation (VA-ECMO), and the Impella-series of percutaneous left ventricular assist devices or, more commonly, pumps (pLVAD; Danvers, MA, USA) [4,5]. 

Although these devices are widely used in clinical practice, available evidence on their use and efficacy is constantly evolving. For instance, the clinical guidelines do not recommend the use of IABP for the treatment of CS and no longer recommend it for support in high-risk PCI procedures [4,6]. Additionally, they are often assessed independently in clinical studies, rather than comparatively, with available observational studies [3,7,8] and literature reviews [8,9,10,11,12,13,14,15,16,17] focusing on single devices. Available reviews only compare Impella with IABP for the treatment of CS [18], while works comparing Impella with VA-ECMO are more focused on other diseases, such as acute right ventricle failure [19]. Furthermore, published studies typically addressed a limited set of outcomes, especially mortality [2,20]. No ongoing or recently completed review comparing Impella with VA-ECMO in patients with CS can be found in PROSPERO, a register for prospective reviews. 

Comparative effectiveness data have become even more relevant since the new Health Technology Assessment Regulation (HTAR 2021/2282) [21] was approved in December 2021 by the EU Parliament. With this regulation, high-risk, life-saving technologies would need to be comparatively assessed at the European level, to provide each Member State with robust clinical and socio-economic evidence in support of national coverage and reimbursement. 

The aim of this work was to perform a comprehensive systematic review on the use of Impella against VA-ECMO in the treatment of CS, considering not only major clinical outcomes, but also quality of life (QoL) and economic resources used. 

## 2. Materials and Methods

Our systematic literature review followed the methodological guidance for systematic reviews and meta-analyses [22] and the MOOSE reporting guidelines for meta-analyses [23]. The study protocol was registered on PROSPERO (ID: 323805). The 27-item PRISMA checklist is reported in the Supplementary Materials (Table S1). 

The search was performed in two electronic databases, MEDLINE and WEB OF SCIENCE. Relevant articles were searched from 2017 to 2022. This timeframe was set to account for the most recent use of Impella and VA-ECMO technologies in clinical practice to treat patients with CS. Additionally, the references of the most pertinent published literature reviews were screened, and other relevant papers were identified (snowballing). The search strategy was defined with an iterative approach. A preliminary, exploratory search was performed by three researchers individually (VA, LS, CR), relevant keywords were discussed among the research team, and the final query was fine-tuned with the support of the clinicians (AMS, MP). The search was conducted on 21 February 2022, and was restricted to title and abstract in PUBMED, and to topic (title, abstract, and keywords) in WEB OF SCIENCE. Additional details of the search string can be found in Table S2. 

We included randomized controlled trials (RCTs), observational studies, both prospective and retrospective, and economic evaluations (e.g., cost-effectiveness analyses or budget impact analyses). Other designs, including case reports, single-patient case studies, guidelines, protocols, position papers, consensus statements, editorials, and letters were excluded. Only studies written in English or Italian were included for data synthesis. Only studies individually focused on Impella or VA-ECMO were eligible. Articles on the treatment of CS with devices other than Impella or VA-ECMO (e.g., IABP) or with a combination of devices (e.g., ECPella) were excluded. The target patients for our analyses were adult populations suffering from CS. Detailed information on inclusion criteria is provided in Table S3. 

Two researchers (VA, LS) screened the retrieved articles based on title and abstract. Titles eligible for full-text reading were read independently by VA and LS, and disagreements on final inclusions were resolved by a third researcher (CR). Two MDs (AMS, MP) contributed to the study selection, assessing the characteristics of the patients in the selected studies. The entire research team read all the studies included for analysis. 

Data were extracted using a datasheet in Microsoft Excel, and included: (i) an overview of the studies (e.g., country, year, study design); (ii) patient information (e.g., patient characteristics, sample size, mean age); (iii) device information (i.e., device type, Impella model); (iv) outcome information (e.g., survival and cardiac outcomes, safety in hospital complications, device-related outcomes); (v) socio-economic information (i.e., resource use, QoL). The full list of variables extracted is provided in Table S4. 

A descriptive overview provides key summary statistics of the information extracted from the studies. Meta-analyses were performed on the most relevant outcomes, considering separately studies involving VA-ECMO and Impella. The objective was to understand the overall effect size (ES) of each outcome, its significance, and the level of heterogeneity among studies [24]. Several outcomes were considered, including mortality, weaning, bridge to LVAD, bridge to transplant, bleeding, limb ischemia, ischemic stroke, myocardial recovery, renal failure, and days on support, with sub-group analyses based on the timing of measurement. For mortality, a distinction was made between chronological timings (i.e., 30 days, 6 months, 1 year) and qualitative timings (i.e., in hospital, discharge, to next therapy, to explant, on device) to achieve consistent meta-analyses. The other outcomes were primarily measured in hospital. Outcomes were pooled through a random effect meta-analysis of proportions or continuous values. A test on the summary effect measure was given, including a test for heterogeneity, quantified using the I2 metric: the higher the values (from 0% to 100%), the larger the heterogeneity across studies [24,25]. Results were shown using forest plots, which showed the ES of each study with the corresponding CI and the overall ES of all selected studies. In order to compare outcome values between Impella and VA-ECMO groups and to determine whether the differences were statistically significant, the confidence intervals for those groups were compared. If those intervals did not overlap, the difference between groups was considered statistically significant, otherwise the difference was considered conservatively not statistically significant. 

Analyses were performed using STATA software (StataCorp, version 17, College Station, TX, USA). Quality of the studies and risk of bias were assessed using two critical appraisal tools, respectively, for cohort studies [26] and RCTs [27]. 

## 3. Results

### 3.1. Review Profile

**Figure 1:**
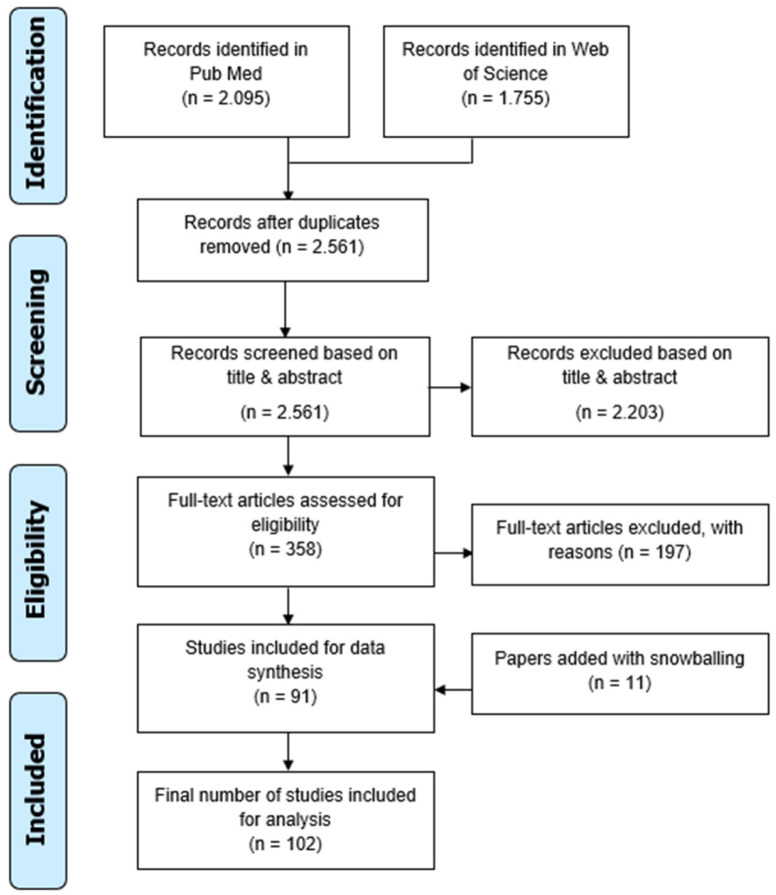
PRISMA flowchart.

From PubMed and Web Of Science, 2095 and 1755 articles were retrieved, respectively, totaling 3850 articles. With RefWorks [28], 1289 duplicates were removed and 2561 records were left for title/abstract screening. After exclusion of 2203 papers based on the title/abstract, a total of 358 articles were read full-text and imported in Zotero [29], a bibliographic reference manager. One-hundred-ninety-six records were excluded after integral reading, and eleven additional papers were included through snowballing. The final selection, therefore, included 102 papers for data analysis. Figure 1 illustrates the PRISMA flowchart [22].

### 3.2. Characteristics of Included Studies

The number of articles published between 2017 and 2022 increased steadily (13% in 2017, 14% in 2018, 17% in 2019, 26% in 2020, 25% in 2021), indicating growing academic attention to the treatment of patients with CS. 

Studies on the treatment of CS with Impella and VA-ECMO are primarily set in Europe (42, 41%) [20,30,31,32,33,34,35,36,37,38,39,40,41,42,43,44,45,46,47,48,49,50,51,52,53,54,55,56,57,58,59,60,61,62,63,64,65,66,67,68,69], followed by North and South America (38, 37%) [2,7,70,71,72,73,74,75,76,77,78,79,80,81,82,83,84,85,86,87,88,89,90,91,92,93,94,95,96,97,98,99,100,101,102,103,104,105], Asia (20, 20%) [3,4,8,106,107,108,109,110,111,112,113,114,115,116,117,118], the Middle East and Africa (2, 2%) [119,120]. The United States is where most of the studies are conducted (35, 34%) [2,7,70,71,72,74,75,76,77,78,79,81,82,83,84,85,86,87,88,89,90,91,92,93,94,96,97,98,99,100,101,102,103,104,105], followed by Germany (19, 19%) [20,33,35,38,39,42,50,53,54,55,57,58,63,64,65,66,67,121,122], Japan (7, 7%) [3,4,69,108,112,115,116], France (6, 6%) [32,34,47,48,52,60], and South Korea (6, 6%) [8,113,114,117,123,124].

Ninety-one (89%) studies are observational studies with a retrospective design [2,3,4,7,8,20,31,32,33,34,36,37,39,40,41,42,43,44,45,46,47,48,49,50,51,52,54,55,56,57,58,59,60,61,64,65,66,68,69,70,71,72,73,74,75,76,77,78,79,80,81,83,84,85,87,88,89,90,91,92,93,94,95,96,97,98,99,100,101,102,103,104,105,106,107,108,109,110,112,113,114,115,116,117,118,119,121,122,123,125,126], while only seven (7%) use prospectively collected data [2,35,53,82,86,111,120,124], and four (4%) use other designs (e.g., RCTs) [30,38,62,63]. Sixty-six (65%) studies are single-centered, whereas thirty-six (35%) are multi-centered. Studies can be single-arm or multi-arm. The former (67, 66%) assess one device only (i.e., Impella or VA-ECMO), whereas the latter have control groups or compare two (27, 26%) or more (8, 8%) devices. The selected studies equally represent Impella and VA-ECMO in the treatment of patients with CS, respectively, 58 (57%), and 44 (43%), with 9 papers focusing on both. Of the Impella studies, 41 (71%) investigate Impella CP, 5 (9%) Impella RP, 25 (43%) Impella 2.5, 20 (34%) Impella 5.0, and 1 (2%) Impella 5.5. 

The average sample size is composed of 304 patients (median 62). The study with the smallest patient sample is conducted on 7 patients [73], while the largest includes 9774 patients [57]. 

Table 1 summarizes how frequently the study outcomes are reported in the selected studies. Mortality is the most frequently recurring outcome (98% frequency), followed by duration of support (59%), and bleeding (58%). Meta-analyses were performed on the clinically relevant outcomes. Study characteristics are summarized in the Supplementary Table S5. 

### 3.3. Evidence from Meta-Analyses

Confidence intervals (CI 95%) were used to assess whether a difference in outcomes between Impella and ECMO is significant because p-values are not available in single-arm analyses. Overlaps between CIs indicate that the difference between devices is not statistically significant. 

#### 3.3.1. Mortality

**Figure 2:**
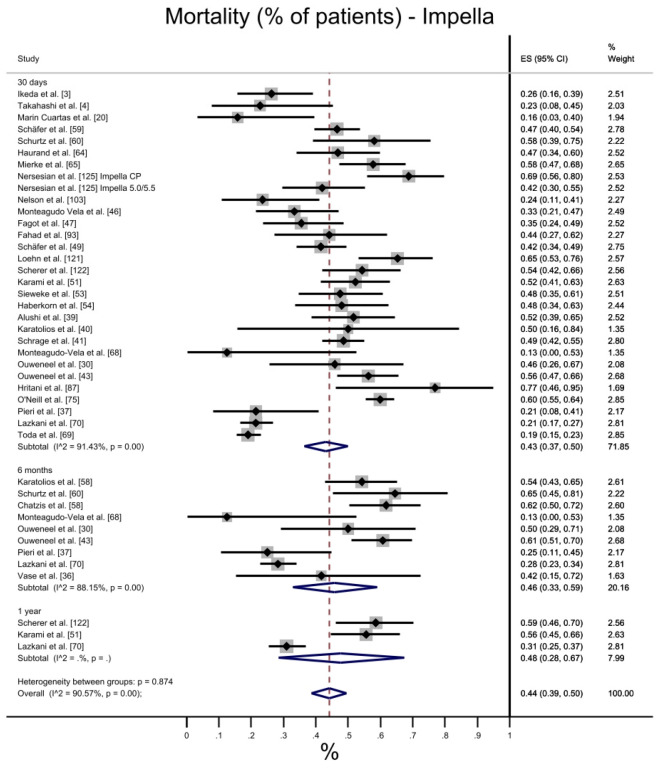

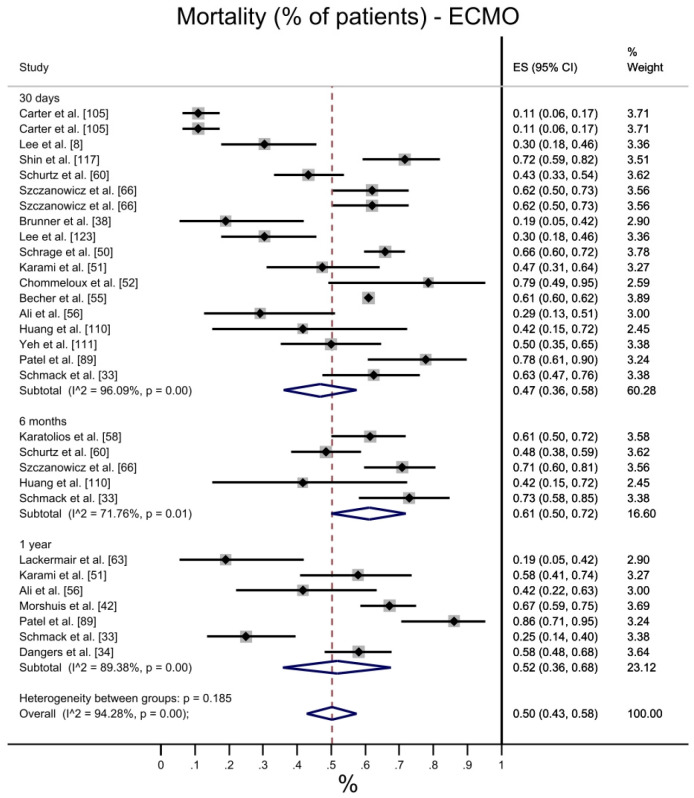
Mortality: Impella vs. VA-ECMO [3,4,8,20,30,33,34,36,37,38,39,40,41,42,43,46,47,49,50,51,52,53,54,55,56,58,59,60,63,64,65,66,68,69,70,75,87,89,93,103,105,110,111,117,121,122,123,125].

Overall mortality (both at 30 days, 6 months, 1 year) was 44% (CI 95%: 39–50%) in patients treated with Impella and 50% (CI 95%: 43–58%) in patients treated with VA-ECMO (Figure 2). Heterogeneity between groups was not statistically significant, and overall heterogeneity was higher for VA-ECMO (I2 = 94.28% vs. 90.57%). More favorable mortality trends for Impella were observed in any timepoints (43% vs. 47% at 30 days; 46% vs. 61% at 6 months; 48% vs. 52% at 1 year). With the data extracted from different studies for the considered time-points, we can highlight a non-coherent higher mortality rate for VA-ECMO at 6 months (61%) compared to the same datum at 1 year (52%). 

As for qualitatively labelled mortality outcomes (i.e., at discharge, in hospital, to the next therapy, to explant, and on device), overall mortality showed a 45% ES (CI 95%: 40–49%) for Impella, compared with 49% (CI 95%: 45–53%) for VA-ECMO. Heterogeneity between groups was statistically significant for Impella and VA-ECMO treatments, with similar heterogeneity (I2). However, this difference is not maintained across all sub-group mortalities. For instance, mortality at discharge was 44% in Impella patients and 54% in VA-ECMO, whereas the opposite is observed when on device mortality is considered (57% vs. 35%) (Figure S1). 

#### 3.3.2. Weaning

**Figure 3:**
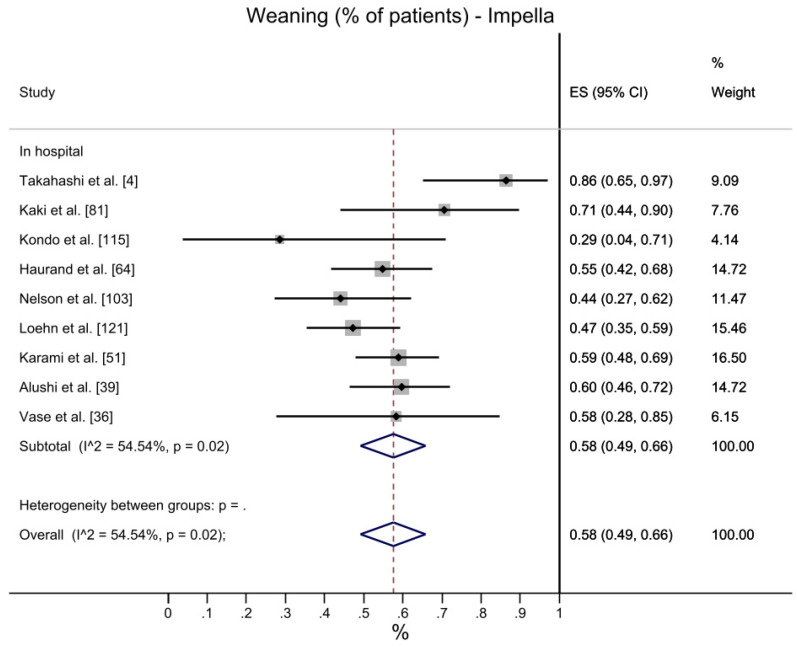

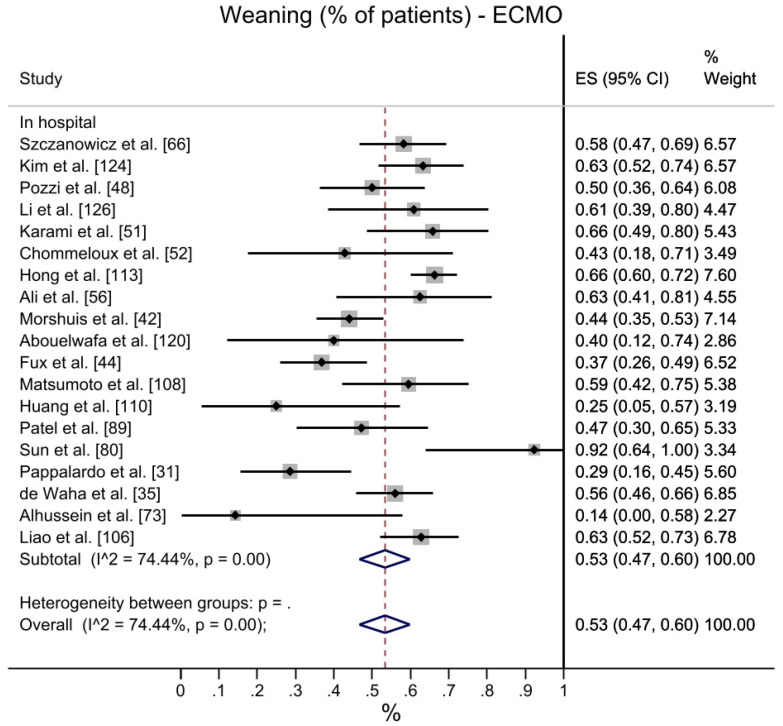
Weaning: Impella vs. VA-ECMO. [4,31,35,36,39,42,44,48,51,52,56,64,66,73,80,81,89,103,106,108,110,113,115,120,121,124,126].

A total of 58% of the population is successfully weaned from Impella (CI 95%: 49–66%), with a similar value for VA-ECMO (53%, CI 95%: 47–60%), although heterogeneity is higher for VA-ECMO (Figure 3), and this difference is not statistically significant (overlaps in the CI are observed).

#### 3.3.3. Bleeding

**Figure 4:**
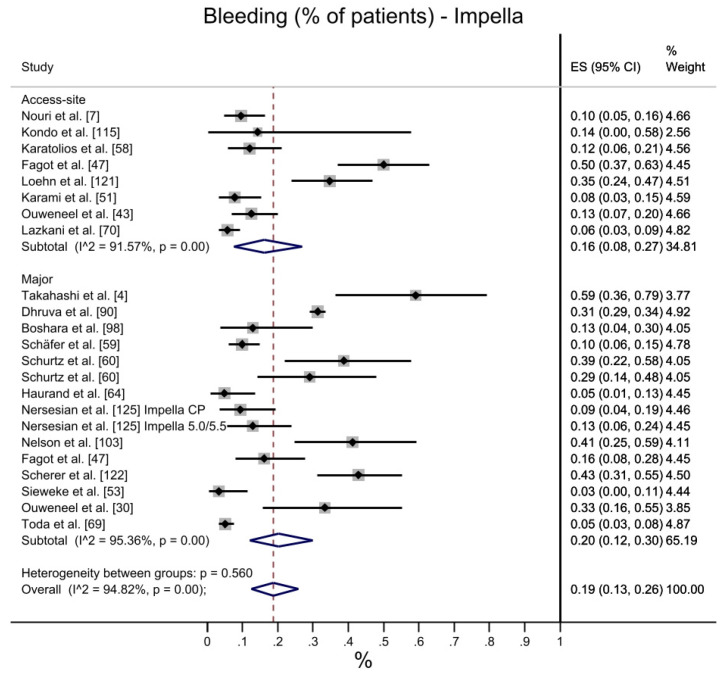

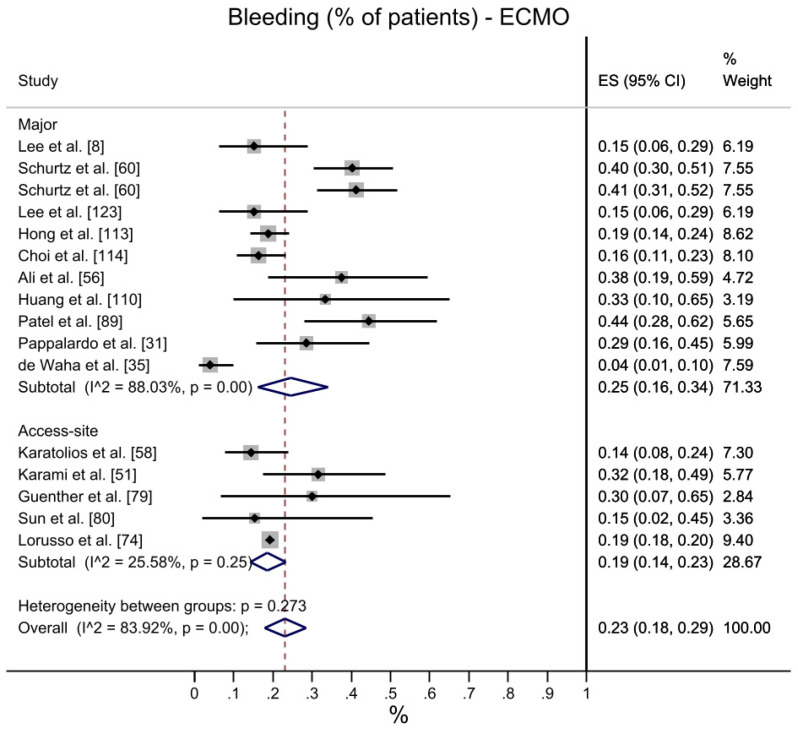
Bleeding: Impella vs. VA-ECMO. [4,7,8,30,31,35,43,47,51,53,56,58,59,60,64,69,70,74,79,80,89,90,98,103,110,113,114,115,121,122,123,125].

Bleeding was measured with diverse scales in the selected studies (e.g., GUSTO, BARC). To ensure outcome comparability, only access-site bleeding and major bleeding (or equivalent) were assessed. Overall bleeding reported a 19% ES for Impella-treated patients, and 23% for VA-ECMO. Access-site bleeding showed a 16% ES for Impella and 19% for VA-ECMO, whereas the percentage of patients experiencing major bleeding was 20% in the former group and 25% in the latter. In general, heterogeneity was higher for the Impella arm, although this lacks statistical significance (Figure 4).

#### 3.3.4. Limb Ischemia

**Figure 5:**
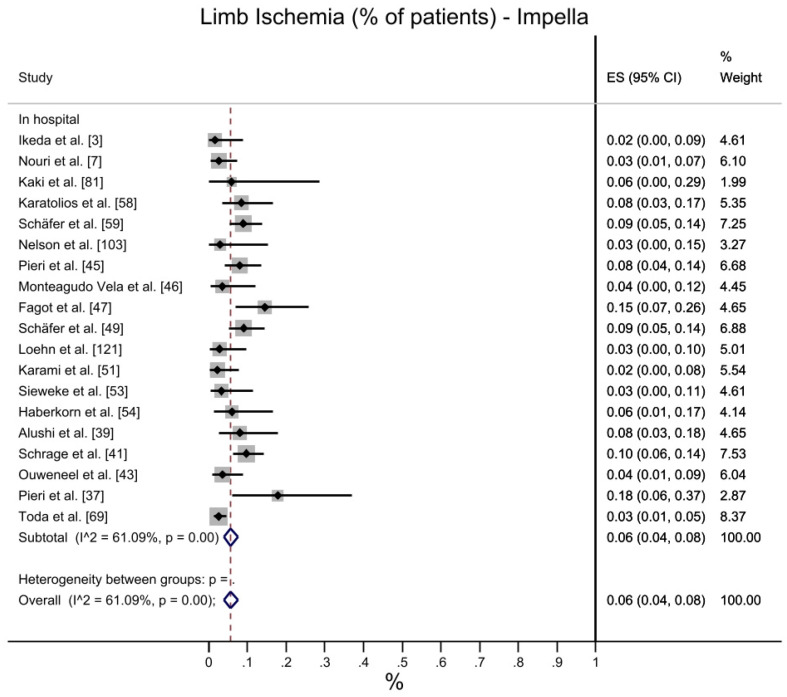

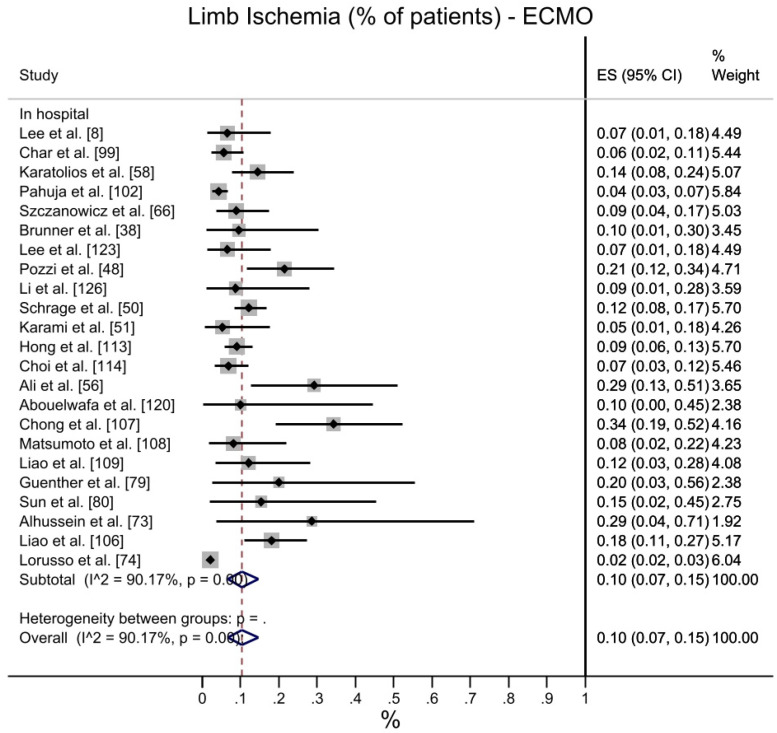
Limb Ischemia: Impella vs. VA-ECMO. [3,7,8,37,38,39,41,43,45,46,47,48,50,51,53,54,56,58,59,66,69,73,74,79,80,81,99,102,103,106,107,108,109,113,114,120,121,123,126].

A total of 6% (CI 95%: 4–8%) of patients treated with Impella reported limb ischemia, compared to 10% (CI 95%: 7–15%) for VA-ECMO; the latter showed higher heterogeneity (I2 = 90.17%) compared with Impella (I2 = 61.09%). The observed data suggest that limb ischemia complications occur less frequently in populations treated with Impella (Figure 5).

#### 3.3.5. Ischemic Stroke

**Figure 6:**
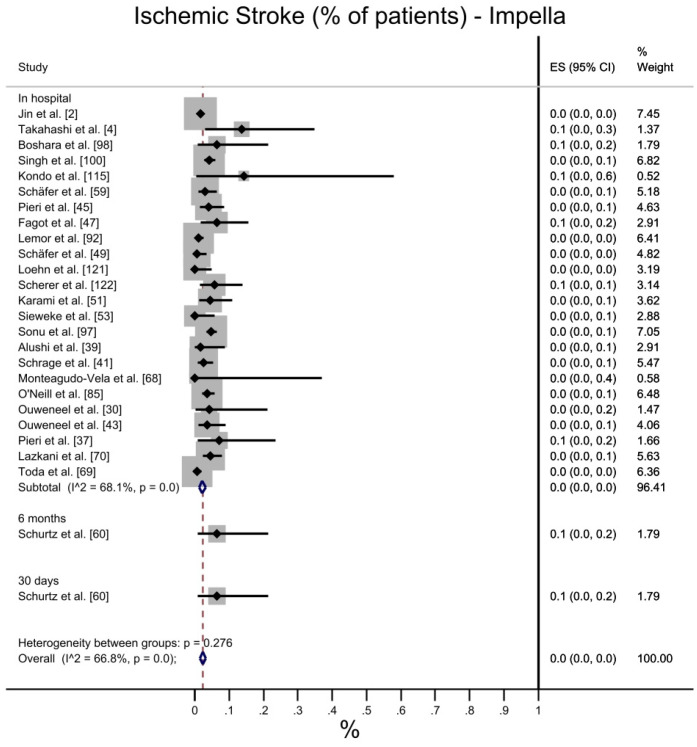

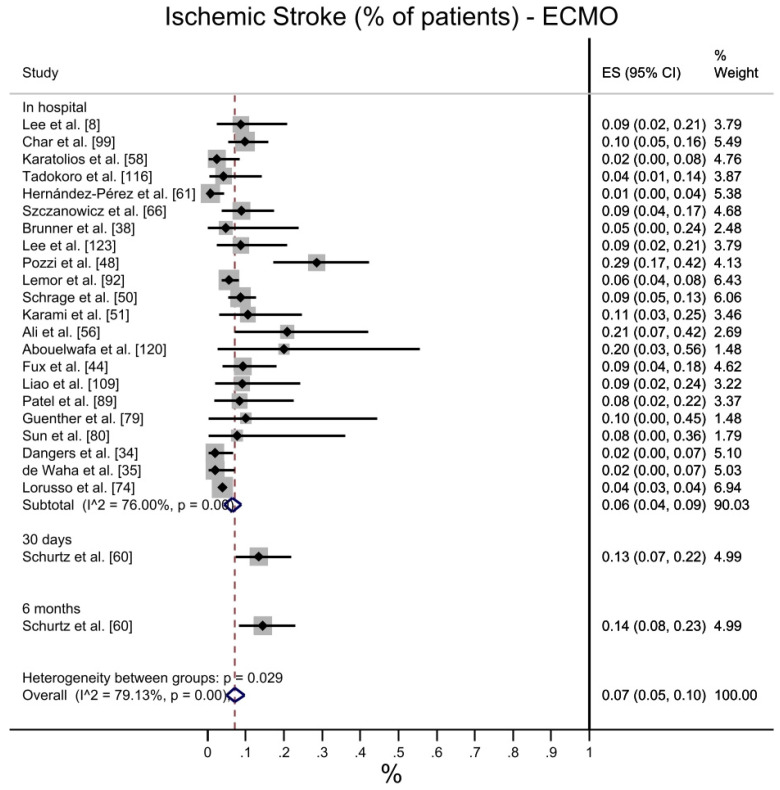
Ischemic stroke: Impella vs. VA-ECMO. [2,4,8,30,34,35,37,38,39,41,43,44,45,47,48,49,50,51,56,58,59,60,61,66,68,69,70,74,79,80,85,89,92,97,98,99,100,109,116,120,121,122,123].

Ischemic stroke is more likely to occur in patients treated with VA-ECMO (7%, with CI 95%: 5–10%) than with Impella, with a 0% observed ES (Figure 6). Despite the higher heterogeneity observed for VA-ECMO, the difference was statistically significant, both in pooled values and in all the measurements considered (in hospital, at 30 days, 6 months). These ESs were computed including different ischemic stroke measurements (i.e., in hospital, at 30 days, 6 months), but results are also confirmed when only equivalent measurements are considered. 

#### 3.3.6. Bridge to LVAD

The percentage of patients bridged to long-term LVAD is the same for Impella and VA-ECMO groups (10%), without statistically significant differences (Figure S2).

#### 3.3.7. Bridge to Transplant

The percentage of patients bridged to heart transplant is 4% (CI 95%: 0–12%) in Impella, and 6% (CI 95%: 3–9%) in VA-ECMO, although the difference is not statistically significant (Figure S3).

#### 3.3.8. Renal Failure

Renal failure events had a similar frequency for the populations treated with Impella (34% ES, CI 95%: 26–43%) and with VA-ECMO (38% ES, CI 95%: 31–42%), although this lacks statistical significance. High level of overall heterogeneity was reported for both devices (Figure S4).

#### 3.3.9. Days on Support

Days on support were reported either with standard deviation or interquartile range. The width of the interquartile range was approximated, computed by multiplying standard deviation by 1.35. This approximation can be applied with large sample sizes and outcome distribution similar to the normal distribution [127]. It is a plausible approximation, given that the same population is described and treated with different devices. Overall, a longer support duration is observed for VA-ECMO (4.47 days, CI 95%: 2.93–6.00), compared to Impella (3.23 days, CI 95%: 1.99–4.48) (Figure S5).

### 3.4. Risk of Bias Assessment

Risk of bias was assessed using tools specifically designed for cohort studies and RCTs [26,27]. In general, the selected studies perform well in terms of risk of bias, scoring “Yes” in the majority of fields on the checklists. All the RCTs highlighted a lack of being blinded to treatment assignment; however, this is obvious in RCTs on devices, as it is impossible to create the equivalent of a placebo for a device implantation or to mask VA-ECMO or Impella. Given the low potential bias resulting from this analysis, all studies were maintained for inclusion. Results from risk of bias assessment are reported in Tables S6 and S7.

## 4. Discussion

This systematic review and meta-analyses aimed at framing the comparison between Impella and VA-ECMO in patients with CS. Previous literature reviews were mostly focused on a limited set of outcomes, such as mortality or bleeding. In an effort to extend the available literature in the field, the present review investigates a wider, more comprehensive range of outcomes, namely, mortality, bleeding, weaning, bridge to LVAD, bridge to transplant, myocardial recovery, limb ischemia, renal failure, ischemic stroke, and days on support. The outcomes studied in the current work are crucial not only from a clinical standpoint but also because they inform the development of future economic evaluations. Performing economic evaluations of medical devices is challenging due to their distinctive features compared to drugs, including, but not limited to, incremental innovation, dynamic pricing, the learning curve, and dependence on organizational factors. These differences need to be taken into consideration when conducting evaluation [128,129,130,131]. 

To date, several meta-analyses evaluated MCS devices for the management of patients with CS. Vargas and colleagues investigated pooled estimates of 30-day mortality and complications of Impella devices against IABP or medical treatment, specifically by evaluating short-term mortality, stroke, major bleeding, and peripheral ischemic complications [14]. Batsides et al. [15] investigated Impella 5.0 devices only, by summarizing survival outcomes and complications (i.e., stroke, bleeding, limb ischemia, hemolysis). Iannaccone et al. [10] performed a meta-analysis of observational studies reporting 30-day mortality for Impella. 

To our knowledge, there are few comparative meta-analyses on Impella versus VA-ECMO. The meta-analysis performed by Affass and colleagues [19] focused on acute right ventricle failure, and its results favored VA-ECMO, despite a lack of statistical significance. Batchelor et al. assessed the two devices through six studies focused on acute myocardial infarction CS and found that Impella was associated with a reduced risk of in-hospital and medium-term mortality as compared to VA-ECMO [132]. Ahmad et al. compared 13 studies and found that Impella use was associated with lower incidence of in-hospital mortality, stroke, access-site bleeding, major bleeding, and limb ischemia, although patients in the ECMO group had higher baseline lactate levels [133]. Lastly, Abusnina et al. found that the use of Impella is associated with lower rates of in-hospital mortality, bleeding, and stroke compared to VA-ECMO, although the need to conduct future randomized studies with adequate sample sizes is advocated [134].

In this context of limited comparative evidence among MCS devices, by combining a larger set of outcomes and data available in different study designs, from the rigorous data of RCTs to the real-world data of observational studies [131], the present study contributes to expanding the body of evidence in a research topic that is currently under the lens of researchers and clinicians, as demonstrated by the growing number of published papers over time. The meta-analyses performed as part of this work allowed for the synthesis and interpretation of the data from the selected studies with a comparative perspective. Based on observed ESs, Impella seems to be associated with better results in mortality, bleeding, weaning, limb ischemia, renal failure, and ischemic stroke, as well as fewer days on support. However, these results are not statistically significant, and therefore need to be interpreted with caution. The only statistically significant result refers to ischemic stroke, which confirms with a sufficient degree of statistical confidence that patients suffering from CS who are treated with Impella are less likely to experience ischemic strokes compared to patients treated with VA-ECMO. This evidence is impressive, as ischemic strokes have long-term impacts on patients, especially in terms of QoL and general well-being after discharge. Mortality outcomes (at 30 days and at discharge) were significant when pre-PCI applications of Impella were compared against post-PCI applications, favoring the former (Figures S6 and S7). Although these results are informative for clinical practice, they are neutral with respect to the factors contributing to the selection of devices that present different peculiarities. For example, Impella pumps provide for the unloading of the left ventricle, but do not support the right ventricle or gas exchange in cases of pulmonary oedema with impaired lung function. VA-ECMO alone, on the contrary, provides biventricular circulatory support, but not unloading of the left ventricle. Strategies of combined support are also feasible to overcome limitations of single device, but, in line with the study design and methodology, this population of patients was not included in the present analysis. 

This review also aimed at mapping and comparing socio-economic endpoints. However, virtually no publication among the selected papers assesses either QoL or the resource use associated with both devices. While this is important per se, it becomes particularly relevant when considering the increasing need to perform comparative economic analyses of health technologies to support their adoption. Based on the provisions from the new HTA Regulation (HTAR 2021/2282) [21] in the EU, as well as the national guidelines of most European countries, funding and reimbursement decisions need to be linked to the evidence from economic analyses [135,136,137]. Conducting both economic evaluations (e.g., cost-effectiveness analysis or cost-utility analysis) and broader health technology assessments will become increasingly pivotal for the uptake of new health technologies and their coverage under national health services. This is key in allowing comparative assessments of MCS technologies by considering equally the financial burden for government budgets and the health impact on patient outcomes. Filling this gap will be not only economically relevant, but also regulatorily necessary in the near future.

This work has several limitations. First, it investigated Impella and VA-ECMO as two alternative treatment courses for patients suffering from CS. However, it does not provide an exhaustive synthesis of the published evidence on the treatment for CS, as studies focused on combinations of devices (i.e., ECPella) were excluded. Furthermore, this work includes heterogeneous studies per country and publication year that might reflect heterogeneous populations. Although studies were included only if they met rigorous criteria, the variability in clinical practice across countries could contribute to the heterogeneity between groups observed in the meta-analyses. Recently introduced classifications in CS, such as the SCAI Shock Stage [138], could not be used to categorize the selected studies, as they were not systematically available. Moreover, the nature of CS treatments, associated with frequently changing clinical pathways and rapidly escalating severity levels in patients [138,139], might hamper the quality of the stratification of the target population. Studies were selected based on the year of publication, regardless of the year of data collection. While this is common in literature reviews, it might reflect patient populations hospitalized over different time windows and treated according to different clinical practices. Another limitation refers to the outcome set considered. While the aim of this work was to perform an outcome analysis as comprehensive as possible in the treatment of CS, certain endpoints (e.g., ventricular injuries) could not be included in the meta-analyses, as there were not enough observations compared to the most common measures. Most studies used retrospective data, which could limit the comparability of the outcomes due to missing observations or potentially different ways to interpret the study data in retrospect. Similarly, the vast majority of the studies are observational studies. While this is common in the area of mechanical support [140], significant residual confounders could influence the outcomes being studied, as opposed to when rigorous data from study designs such as RCTs are used. Lastly, only papers published in English were included, excluding possible relevant studies on CS in other languages.

## 5. Conclusions

This review highlighted the need to conduct more comparative studies in the field of MCS health technologies for the treatment of cardiogenic shock. Despite the lack of conclusive results for the other outcomes evaluated, the study highlighted possible areas in which further data collection is needed. The results provided are also useful for the development of future cost-effectiveness and budget impact analyses, as the management of patients and their complications lead to an economic burden for the national healthcare service and for the society as a whole. Comparative analyses in terms of both clinical and economic outcomes may provide further evidence to support the stakeholders in the optimization of management strategies for patients with CS. 

## Figures and Tables

**Table 1:** Clinical outcomes extracted from selected papers.

Outcomes	N	%	Included in Meta-Analysis
Mortality	100	98%	Yes
Bleeding	60	59%	Yes
Duration of support	60	59%	Yes
Ischemic stroke	50	49%	Yes
Renal failure	44	43%	Yes
Limb ischemia	40	39%	Yes
Bridge to LVAD	30	29%	Yes
Weaning	27	26%	Yes
Days in hospital/ICU	25	25%	No
Bridge to transplant	23	23%	Yes
Hemolysis	22	22%	No
Sepsis	20	20%	No
Myocardial recovery	10	10%	Yes
Major device malfunction	8	8%	No
Average Impella pump flow	6	6%	No
Device exchange	5	5%	No
Resource use	5	5%	No
Aortic valve injury	3	3%	No
Mitral valve injury	3	3%	No
LV perforation	0	0%	No
Aortic dissection	0	0%	No
Early mobilization and physiotherapy	0	0%	No
Average Impella performance level	0	0%	No
Quality of life	0	0%	No

## Data Availability

This work is a literature review and meta-analysis, i.e., a data synthesis of already available data.

## Supplementary Materials

The following supporting information can be downloaded at: https://www.mdpi.com/article/10.3390/jcdd10040158/s1, Table S1: PRISMA Checklist; Table S2: Search string; Table S3: Inclusion Criteria; Table S4: List of extracted variables; Table S5: Summary of study characteristics; Figure S1: Qualitatively labelled mortality: Impella vs. VA-ECMO; Figure S2: Bridge to LVAD: Impella vs. VA-ECMO; Figure S3: Bridge to transplant: Impella vs. VA-ECMO; Figure S4: Renal failure: Impella vs. VA-ECMO; Figure S5: Days on support: Impella vs. VA-ECMO; Tables S6 and S7: Risk of bias assessment. Figure S6. Pre-PCI vs. Post-PCI mortality at 30 days. Figure S7. Pre-PCI vs. Post-PCI Mortality at discharge.
