# Supplementary material for: Impella Versus VA-ECMO for Patients with Cardiogenic Shock: Comprehensive Systematic Literature Review and Meta-Analyses
Source: J Cardiovasc Dev Dis. 2023 Apr 5;10(4):158. doi: 10.3390/jcdd10040158 (PMC10142129; doi:10.3390/jcdd10040158)
Supplement: Supplementary file 1 [file jcdd-10-00158-s001.zip › jcdd-2280489-supplementary.pdf]

Supplementary materials

Table S1. PRISMA Checklist

SECTION	ITEM	PRISMA – ScR CHECKLIST ITEM	REPORTED ON PAGE #
TITLE			
Title	1	Identify the report as a systematic review, meta-analysis, or both.	1
ABSTRACT			
Structured summary	2	Provide a structured summary including, as applicable: background; objectives; data sources; study eligibility criteria, participants, and interventions; study appraisal and synthesis methods; results; limitations; conclusions and implications of key findings; systematic review registration number.	2
INTRODUCTION			
Rationale	3	Describe the rationale for the review in the context of what is already known.	3
Objectives	4	Provide an explicit statement of questions being addressed with reference to participants, interventions, comparisons, outcomes, and study design (PICOS).	4
METHODS			
Protocol and registration	5	Indicate if a review protocol exists, if and where it can be accessed (e.g., Web address), and, if available, provide registration information including registration number.	4
Eligibility criteria	6	Specify study characteristics (e.g., PICOS, length of follow-up) and report characteristics (e.g., years considered, language, publication status) used as criteria for eligibility, giving rationale.	4-5
Information sources	7	Describe all information sources (e.g., databases with dates of coverage, contact with study authors to identify additional studies) in the search and date last searched.	4
Search	8	Present full electronic search strategy for at least one database, including any limits used, such that it could be repeated.	4
Study selection	9	State the process for selecting studies (i.e., screening, eligibility, included in systematic review, and, if applicable, included in the meta-analysis).	5
Data collection process	10	Describe method of data extraction from reports (e.g., piloted forms, independently, in duplicate) and any processes for obtaining and confirming data from investigators.	5
Data items	11	List and define all variables for which data were sought (e.g., PICOS, funding sources) and any assumptions and simplifications made.	5-6
Risk of bias in individual studies	12	Describe methods used for assessing risk of bias of individual studies (including specification of	6

		whether this was done at the study or outcome level), and how this information is to be used in any data synthesis.	
Summary measures	13	State the principal summary measures (e.g., risk ratio, difference in means).	5
Synthesis of results	14	Describe the methods of handling data and combining results of studies, if done, including measures of consistency (e.g., I^2) for each meta-analysis.	5
Risk of bias across studies	15	Specify any assessment of risk of bias that may affect the cumulative evidence (e.g., publication bias, selective reporting within studies).	10
Additional analyses	16	Describe methods of additional analyses (e.g., sensitivity or subgroup analyses, metaregression), if done, indicating which were pre-specified	NA
RESULTS			
Study selection	17	Give numbers of studies screened, assessed for eligibility, and included in the review, with reasons for exclusions at each stage, ideally with a flow diagram.	6
Study characteristics	18	For each study, present characteristics for which data were extracted (e.g., study size, PICOS, follow-up period) and provide the citations.	6-7
Risk of bias within studies	19	Present data on risk of bias of each study and, if available, any outcome-level assessment (see Item 12).	NA
Results of individual studies	20	For all outcomes considered (benefits or harms), present, for each study: (a) simple summary data for each intervention group and (b) effect estimates and confidence intervals, ideally with a forest plot.	7-10
Synthesis of results	21	Present results of each meta-analysis done, including confidence intervals and measures of consistency.	7-10
Risk of bias across studies	22	Present results of any assessment of risk of bias across studies (see Item 15). Additional analysis 23 Give results of additional analyses, if done (e.g., sensitivity or subgroup analyses, meta-regression [see Item 16]).	10
DISCUSSION			
Summary of evidence	24	Summarize the main findings including the strength of evidence for each main outcome; consider their relevance to key groups (e.g., health care providers, users, and policy makers).	10-12
Limitations	25	Discuss limitations at study and outcome level (e.g., risk of bias), and at review level (e.g., incomplete retrieval of identified research, reporting bias).	12-13
Conclusions	26	Provide a general interpretation of the results in the context of other evidence, and implications for future research.	13-14
FUNDING			

Funding	27	Describe sources of funding for the systematic review and other support (e.g., supply of data); role of funders for the systematic review.	15

Table S2. Search strategy

	Pub Med	Web of Science
Search date	21/02/2022	21/02/2022
Search query	(("Impella") OR ("ECMO" OR "extracorporeal membrane oxygenation" OR "extra-corporeal membrane oxygenation")) AND ("Cardiogenic shock*" OR "Myocardial infarction*" OR "Cardiac shock*" OR "Cardiovascular shock*" OR "Heart shock*" OR "Acute cardiac failure*" OR "Acute decompensated heart failure*" OR "ADHF*" OR "Acute heart insufficiency*" OR "Low cardiac output*" OR "Low output syndrome*" OR "Systolic dysfunction*" OR "Temporary mechanical support*" OR "Extra corporeal support*"OR "Extra-corporeal support*")	(("Impella") OR ("ECMO" OR "extracorporeal membrane oxygenation" OR "extra-corporeal membrane oxygenation")) AND ("Cardiogenic shock*" OR "Myocardial infarction*" OR "Cardiac shock*" OR "Cardiovascular shock*" OR "Heart shock*" OR "Acute cardiac failure*" OR "Acute decompensated heart failure*" OR "ADHF*" OR "Acute heart insufficiency*" OR "Low cardiac output*" OR "Low output syndrome*" OR "Systolic dysfunction*" OR "Temporary mechanical support*" OR "Extra corporeal support*"OR "Extra-corporeal support*")
Field	Title/Abstract	All field (Topic)
Time frame	2017-2022	2017-2022

Table S3. Inclusion and exclusion criteria

	Inclusion	Exclusion
Study Design	• Randomized control trials (RCTs), Observational studies (e.g., prospective, retrospective)<br>• Economic evaluations (e.g., cost-effectiveness analyses, budget impact analyses)	• Case reports<br>• Case studies on single patients, clinical guidelines and procedures<br>• Diagnostic procedures<br>• Study protocols<br>• Reviews, systematic literature reviews, meta-analyses<br>• Position papers<br>• Expert consensus statement<br>• Editorials<br>• Comments and letters
Other characteristics	• Specific outcome values<br>• Data disaggregated by device	• Aggregated outcomes<br>• No data<br>• Text not available
Language	• English<br>• Italian	Other languages
Device	• Impella<br>• ECMO	• Combination of ECMO and Impella (ECPella)

		• Other devices (IABP, TandemHeart, Centrimag, …)<br>• Pharmacologic therapies
Target Population	Cardiogenic shock	• Cardiac arrest<br>• Post cardiotomy<br>• Protected PCI<br>• Refractory cardiogenic shock<br>• Takotsubo cardiomyopathy<br>• viral or bacteriological infections (e.g., COVID)<br>• Other diseases (pulmonary, cerebral, …)
Age	Adults (except for pregnant women)	Children, adolescents

Table S4. Data extraction: list of variables

Classification	Outcome	Unit of Measure
Outcome - Survival and cardiac outcomes	Mortality:<br>- quantitative timings: 30 days; 6 months, 1 year<br>- qualitative timings: discharge, on device, to next therapy, in hospital, to explant	Number of Patients
	Survival (idem as mortality)	Number of Patients
	Weaning	Number of Patients
	Myocardial recovery	Number of Patients
	Bridge to LVAD	Number of Patients
	Bridge to transplant	Number of Patients
Outcome - Safety in hospital complications	Bleeding (major, access-site)	Number of Patients
	Limb ischemia	Number of Patients
	Ischemic stroke	Number of Patients
	LV perforation	Number of Patients
	Aortic valve injury	Number of Patients
	Mitral valve injury	Number of Patients
	Aortic dissection	Number of Patients
	Hemolysis (major, minor)	Number of Patients
	Sepsis	Number of Patients
	Renal failure (acute, ...)	Number of Patients
	Other outcomes	Number of Patients
Outcome - Device related outcomes	Days in hospital/ICU	Days
	Duration of support (days on ECMO/Impella)	Days
	Early mobilization and physiotherapy	Number of Patients
	Average Impella pump flow	l/min
	Average Impella performance level	0-9

	Major device malfunction	Number of events
	Device exchange	Number of events
Resource use and Quality of life	Resource use	Currency
	Quality of life	EuroQoL

Table S5. Characteristics of included studies

Author	Country	Study design (RCT, Observational study, …)	Device	N. Patients	Average age
Jin (2022)	United States	Retrospective observational multi-centre study (database)	Impella	1592	66
Ikeda (2022)	Japan	Retrospective observational single-centre study	Impella	61	68
Nouri (2022)	United States	Retrospective observational single-centre study	Impella	115	64
Takahashi (2022)	Japan	Retrospective observational single-centre study	Impella	22	74
Marin Cuartas (2022)	Germany	Retrospective observational single-centre study	Impella	19	65
Carter (2022)	United States	Retrospective observational multi-centre study (database)	ECMO	146	33
Lee (2021)	South Korea	Retrospective observational multi-centre study (database)	ECMO	269	62
Dhruva (2020)	United States	Retrospective observational multi-centre study (database)	Impella	1680	64
Boshara (2021)	United States	Retrospective observational single-centre study	Impella	31	64
Kaki (2019)	United States	Retrospective observational single-centre study	Impella	17	68
Lang (2021)	Germany	Retrospective observational multi-centre study	Impella	3945	66
			ECMO	9774	66
Char (2021)	United States	Retrospective observational single-centre study	ECMO	143	58
Singh (2021)	United States	Retrospective observational multi-centre study	Impella	649	65
Hernandez-Montfort (2021)	United States	Retrospective observational multi-centre study	Impella	148	58
			ECMO	106	58
Kondo (2021)	Japan	Retrospective observational single-centre study	Impella	7	43
Karatolios (2021)	Germany	Retrospective observational single-centre study	Impella	83	64
Karatolios (2021)			ECMO	83	63
Schäfer (2021)	Germany; Italy	Retrospective observational multi-centre study	Impella	202	66
Tadokoro (2021)	Japan	Retrospective observational single-centre study	ECMO	48	44
Shin (2021)	South Korea	Retrospective observational single-centre study	ECMO	67	>65
Schurtz (2021)	France	Retrospective observational single-centre study	Impella	31	59
			ECMO	97	52
Pahuja (2021)	United States	Retrospective observational multi-centre study	ECMO	444	
Hernández-Pérez (2021)	Spain	Retrospective observational single-centre study	ECMO	130	52
Karami (2021)	Netherlands	Multi-centre randomized control trial	Impella	24	58

Wang (2021)	China	Retrospective observational multi-centre study (database)	ECMO	235	57
Lackermair (2021)	Germany	Multi-centre randomized control trial	ECMO	21	62
Haurand (2021)	Germany	Retrospective observational single-centre study	Impella	62	>60
Mierke (2021)	Germany	Retrospective observational single-centre study	Impella	97	69
Szczanowicz (2021)	Germany	Retrospective observational single-centre study	ECMO	79	60
Kim (2021)	South Korea	Prospective observational single-centre study	ECMO	79	61
Nersesian (2021)	Germany	Retrospective observational multi-centre study	Impella	64	70
			Impella	62	59
Brunner (2019)	Germany	Multi-centre randomized control trial	ECMO	21	62
Nelson (2021)	United States	Retrospective observational single-centre study	Impella	34	58
Diakos (2021)	United States	Retrospective observational single-centre study	Impella	63	
			ECMO	37	
Pieri (2020)	Italy	Retrospective observational single-centre study	Impella	150	>55
Monteagudo Vela (2020)	United Kingdom	Retrospective observational single-centre study	Impella	57	54
Lee (2020)	South Korea	Retrospective observational single-centre study	ECMO	46	65
Kajy (2020)	United States	Retrospective observational single-centre study	Impella	29	66
Fagot (2020)	France	Retrospective observational single-centre study	Impella	62	58
Pozzi (2020)	France	Retrospective observational single-centre study	ECMO	56	57
Li (2020)	China	Retrospective observational single-centre study	ECMO	23	55
Basir (2019)	United States	Prospective observational multi-centre study	Impella	171	63
Lemor (2020)	United States	Retrospective observational multi-centre study (database)	Impella	450	60
			ECMO	450	61
Esposito (2019)	United States	Retrospective observational single-centre study	Impella	23	62
Fahad (2020)	United States	Retrospective observational single-centre study	Impella	34	>60
Schäfer (2020)	Germany; Denmark	Retrospective observational multi-centre study	Impella	166	65
Hassett (2020)	United States	Retrospective observational single-centre study	Impella	79	63
Schrage (2020)	Germany	Retrospective observational multi-centre study	ECMO	255	57
Loehn (2020)	Germany	Retrospective observational single-centre study	Impella	39	66
Scherer (2020)	Germany	Retrospective observational single-centre study	Impella	70	67
Karami (2020)	Netherlands	Retrospective observational multi-centre study	Impella	90	60

			ECMO	38	55
Chommeloux (2020)	France	Retrospective observational single-centre study	ECMO	14	58
Sieweke (2020)	Germany	Prospective observational single-centre study	Impella	24	64
Hong (2020)	South Korea	Retrospective observational single-centre study	ECMO	255	64
Choi (2020)	South Korea	Retrospective observational single-centre study	ECMO	147	65
Trpkov (2020)	Canada	Retrospective observational single-centre study	Impella	34	57
Haberkorn (2020)	Germany	Retrospective observational single-centre study	Impella	50	65
Becher (2020)	Germany	Retrospective observational multi-centre study (database)	ECMO	8351	62
Vallabhajosyula (2020)	United States	Retrospective observational multi-centre study (database)	ECMO	1469	58
Sonu (2020)	United States	Retrospective observational multi-centre study (database)	Impella	840	69
Ali (2020)	United Kingdom	Retrospective observational single-centre study	ECMO	24	35
Alushi (2019)	Germany	Retrospective observational multi-centre study	Impella	62	73
Yourshaw (2019)	United States	Retrospective observational single-centre study	Impella	25	55
Karatolios (2019)	Greece	Retrospective observational single-centre study	Impella	8	78
Schrage (2019)	Europe	Retrospective observational multi-centre study (database)	Impella	115	71
Morshuis (2019)	Germany	Retrospective observational single-centre study	ECMO	134	53
Monteagudo-Vela (2021)	United Kingdom	Retrospective observational single-centre study	Impella	8	49
O'Neill (2019)	United States	Retrospective observational multi-centre study (database)	Impella	475	65
Garan (2019)	United States	Prospective observational single-centre study	Impella	11	61
			ECMO	16	64
Ouweneel (2017)	Netherlands	Multi-centre randomized control trial	Impella	24	58
Ouweneel (2019)	Netherlands	Retrospective observational single-centre study	Impella	112	60
Hritani (2019)	United States	Retrospective observational single-centre study	Impella	13	74
Abouelwafa (2019)	Egypt	Prospective observational single-centre study	ECMO	10	43
Rohm (2019)	United States	Retrospective observational single-centre study	Impella	204	60
Fux (2019)	Sweden	Retrospective observational single-centre study	ECMO	76	52
O'Neill (2018)	United States	Retrospective observational multi-centre study (database)	Impella	479	65
Chong (2018)	Taiwan	Retrospective observational single-centre study	ECMO	35	41
Matsumoto (2018)	Japan	Retrospective observational single-centre study	ECMO	37	42
El Sibai (2018)	United States	Retrospective observational multi-centre study (database)	ECMO	992	51

Liao (2018)	China	Retrospective observational single-centre study	ECMO	33	33
Basir (2018)	United States	Retrospective observational multi-centre study	Impella	41	65
Huang (2018)	Taiwan	Retrospective observational single-centre study	ECMO	46	57
Yeh (2018)	Taiwan	Prospective observational single-centre study	ECMO	48	57
Esposito (2018)	United States	Retrospective observational single-centre study	Impella	19	60
El Sibai (2018)	Lebanon	Retrospective observational multi-centre study (database)	ECMO	796	50
Patel (2019)	United States	Retrospective observational single-centre study	ECMO	36	63
Guenther (2018)	United States	Retrospective observational single-centre study	ECMO	10	36
Toda (2018)	Japan	Retrospective observational single-centre study	ECMO	32	31
Pieri (2018)	Italy	Retrospective observational single-centre study	Impella	28	66
Sun (2018)	Canada	Retrospective observational single-centre study	ECMO	13	54
Lazkani (2017)	United States	Retrospective observational single-centre study	Impella	81	66
Pappalardo (2017)	Germany; Italy	Retrospective observational multi-centre study	ECMO	42	55
Basir (2017)	United States	Retrospective observational multi-centre study (database)	Impella	287	66
Le Pennec-Prigent (2017)	France	Retrospective observational single-centre study	ECMO	26	52
Schmack (2017)	Germany	Retrospective observational single-centre study	ECMO	28	58
Dangers (2017)	France	Retrospective observational single-centre study	ECMO	105	47
Meraj (2017)	United States	Retrospective observational multi-centre study (database)	Impella	36	70
de Waha (2017)	Germany	Prospective observational multi-centre study	ECMO	100	61
Alhussein (2017)	Canada	Retrospective observational single-centre study	ECMO	7	33
Vase (2017)	Denmark	Retrospective observational single-centre study	Impella	12	63
Liao (2017)	China	Retrospective observational single-centre study	ECMO	94	47
Lorusso (2017)	United States	Retrospective observational multi-centre study (database)	ECMO	5480	53
Toda (2022)	Japan	Retrospective observational multi-centre study (database)	Impella	432	66

Table S6. Risk of bias assessment. Checklist for cohort studies

Study	1	2	3	4	5	6	7	8	9	10	11	12
Jin (2022)	Yes	Yes	Yes	Yes	Yes	No	Yes	Yes	Yes	NA	Yes	Include
Takahashi (2022)	Yes	Yes	Yes	No	NA	No	Yes	Yes	Yes	NA	Yes	Include
Boshara (2021)	NA	NA	Yes	NA	NA	No	Yes	Yes	Yes	NA	No	Include
Char (2021)	Yes	Yes	Yes	No	NA	No	Yes	Yes	Yes	NA	Yes	Include
Singh (2021)	NA	NA	Yes	NA	NA	No	Yes	Yes	Yes	NA	Yes	Include
Karatolios (2021)	Yes	Yes	Yes	No	NA	No	Yes	Yes	Yes	NA	Yes	Include
Schurtz (2021)	Yes	Yes	Yes	No	NA	No	Yes	Yes	Yes	NA	Yes	Include
Nersesian (2021)	Yes	Yes	Yes	No	NA	No	Yes	Yes	Yes	NA	Yes	Include
Monteagudo Vela (2020)	NA	NA	Yes	NA	NA	No	Yes	Yes	Yes	NA	Yes	Include
Lee (2020)	NA	NA	Yes	NA	NA	No	Yes	Yes	Yes	NA	Yes	Include
Loehn (2020)	NA	NA	Yes	NA	NA	No	Yes	Yes	Yes	Yes	Yes	Include
Scherer (2020)	Yes	Yes	Yes	Yes	Yes	No	Yes	Yes	Yes	Yes	Yes	Include
Karami (2020)	Yes	Yes	Yes	Yes	Yes	No	Yes	Yes	Yes	NA	Yes	Include
Sieweke (2020)	NA	NA	Yes	NA	NA	No	Yes	Yes	Yes	NA	Yes	Include
Hong (2020)	NA	NA	Yes	NA	NA	No	Yes	Yes	Yes	NA	Yes	Include
Sonu (2020)	NA	NA	Yes	NA	NA	No	Yes	Yes	Yes	NA	Yes	Include
Alushi (2019)	Yes	Yes	Yes	No	NA	No	Yes	Yes	Yes	NA	Yes	Include
Karatolios (2019)	NA	NA	Yes	NA	NA	No	Yes	Yes	Yes	NA	Yes	Include
Schrage (2019)	Yes	Yes	Yes	Yes	Yes	No	Yes	Yes	Yes	NA	Yes	Include
Monteagudo-Vela (2021)	Yes	Yes	Yes	No	NA	No	Yes	Yes	Yes	NA	Yes	Include
O'Neill (2019)	NA	NA	Yes	NA	NA	No	Yes	Yes	Yes	NA	Yes	Include
Ouweneel (2019)	NA	NA	Yes	NA	NA	No	Yes	Yes	Yes	NA	Yes	Include
Hritani (2019)	NA	NA	Yes	NA	NA	Unclear	Yes	Yes	Yes	NA	Yes	Include
Abouelwafa (2019)	NA	NA	Yes	NA	NA	Unclear	Yes	Yes	Yes	NA	Yes	Include
Fux (2019)	NA	NA	Yes	NA	NA	No	Yes	Yes	Yes	NA	Yes	Include
Chong (2018)	NA	NA	Yes	NA	NA	No	Yes	Yes	Yes	NA	Yes	Include
Liao (2018)	NA	NA	Yes	NA	NA	No	Yes	Yes	Yes	NA	Yes	Include
Toda (2018)	NA	NA	Yes	NA	NA	Unclear	Yes	Yes	Yes	NA	Yes	Include
Pieri (2018)	Yes	Yes	Yes	Yes	No	No	Yes	Yes	Yes	NA	Yes	Include
Sun (2018)	NA	NA	Yes	NA	NA	No	Yes	Yes	Yes	NA	Yes	Include
Dangers (2017)	NA	NA	Yes	NA	NA	No	Yes	Yes	Yes	NA	Yes	Include
Meraj (2017)	NA	NA	Yes	NA	NA	No	Yes	Yes	Yes	NA	Yes	Include

Alhussein (2017)	NA	NA	Yes	NA	NA	Unclear	Yes	Yes	Yes	NA	No	Include
Vase (2017)	NA	NA	Yes	NA	NA	No	Yes	Yes	Yes	NA	Yes	Include
Liao (2017)	NA	NA	Yes	NA	NA	No	Yes	Yes	Yes	NA	Yes	Include
Toda (2022)	NA	NA	Yes	NA	NA	No	Yes	Yes	Yes	NA	Yes	Include

Item 1: Were the two groups similar and recruited from the same population?

Item 2: Were the exposures measured similarly to assign people to both exposed and unexposed groups?

Item 3: Was the exposure measured in a valid and reliable way?

Item 4: Were confounding factors identified?

Item 5: Were strategies to deal with confounding factors stated?

Item 6: Were the groups/participants free of the outcome at the start of the study (or at the moment of exposure)?

Item 7: Were the outcomes measured in a valid and reliable way?

Item 8: Was the follow up time reported and sufficient to be long enough for outcomes to occur?

Item 9: Was follow up complete, and if not, were the reasons to loss to follow up described and explored?

Item 10: Were strategies to address incomplete follow up utilized?

Item 11: Was appropriate statistical analysis used?

Item 12: Overall Appraisal

Table S7. Risk of bias assessment. Checklist for RCTs

**Study**	**1**	**2**	**3**	**4**	**5**	**6**	**7**	**8**	**9**	**10**	**11**	**12**	**13**	**14**
Karami (2021)	Yes	Yes	Yes	Yes	No	Yes	Yes	Yes	Yes	Yes	Yes	Yes	Yes	Include
Brunner (2019)	Yes	Unclear	Yes	Yes	No	Yes	Yes	Yes	Yes	Yes	Yes	Yes	Yes	Include
Ouweneel (2017)	Yes	Yes	Yes	Yes	No	Yes	Yes	Yes	Yes	Yes	Yes	Yes	Yes	Include
Lackermair (2021)	Yes	Yes	Yes	Yes	No	Yes	Yes	Yes	Yes	Yes	Yes	Yes	Yes	Include

Item 1: Was true randomization used for assignment of participants to treatment groups?

Item 2: Was allocation to treatment groups concealed?

Item 3: Were treatment groups similar at the baseline?

Item 4: Were participants blind to treatment assignment?

Item 5: Were those delivering treatment blind to treatment assignment?

Item 6: Were outcomes assessors blind to treatment assignment?

Item 7: Were treatment groups treated identically other than the intervention of interest?

Item 8: Was follow up complete and if not, were differences between groups in terms of their follow up adequately described and analyzed?

Item 9: Were participants analyzed in the groups to which they were randomized?

Item 10: Were outcomes measured in the same way for treatment groups?

Item 11: Were outcomes measured in a reliable way?

Item 12: Was appropriate statistical analysis used?

Item 13: Was the trial design appropriate, and any deviations from the standard RCT design (individual randomization, parallel groups) accounted for in the conduct and analysis of the trial?

Item 14: Overall Appraisal

Figure S1. Mortality in hospital, at discharge, on device, to next therapy, to explant: Impella and VA-ECMO

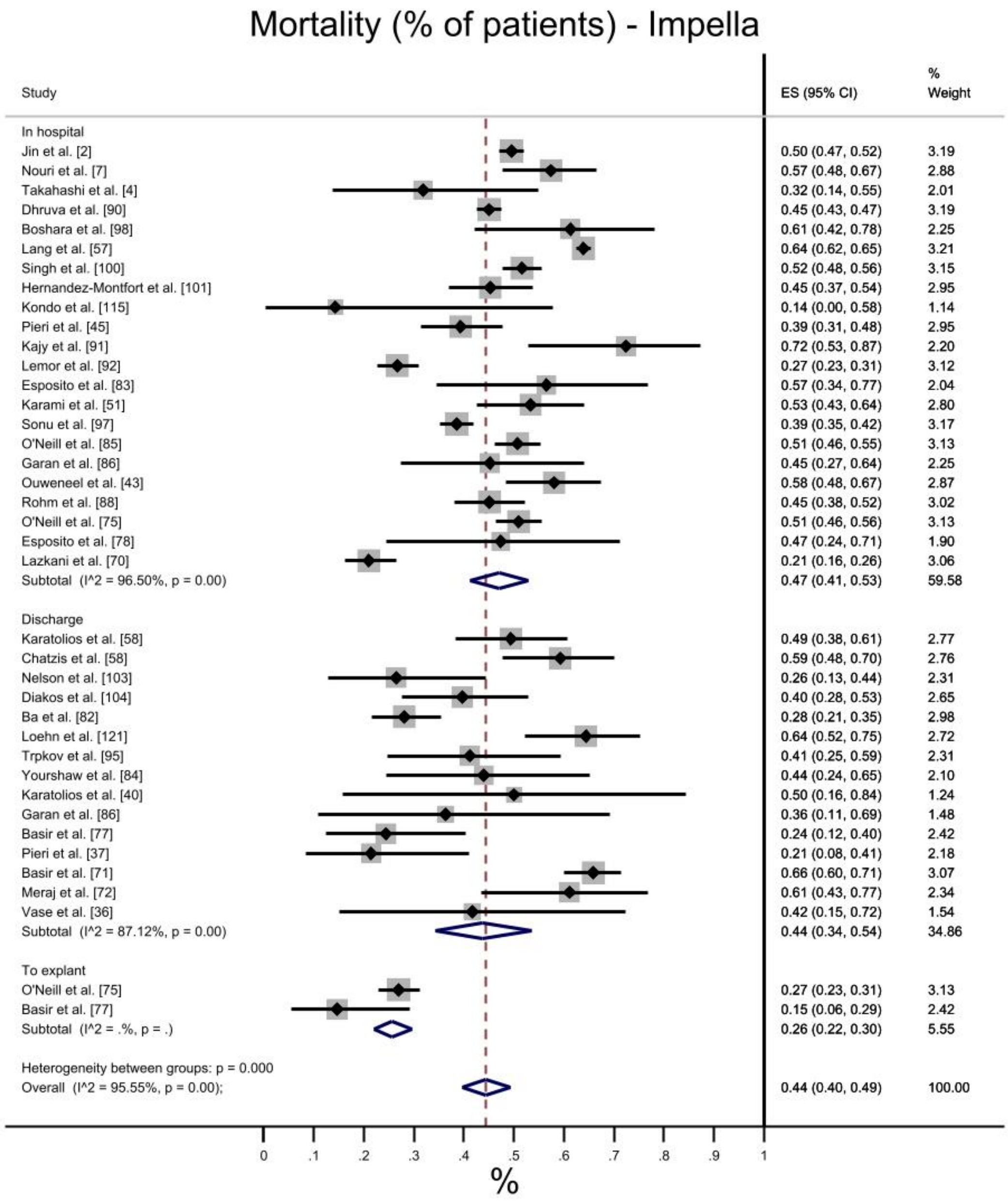
Mortality (% of patients) - Impella
Study
ES (95% CI)
% Weight
In hospital
Jin et al. [2] 0.50 (0.47, 0.52) 3.19
Nouri et al. [7] 0.57 (0.48, 0.67) 2.88
Takahashi et al. [4] 0.32 (0.14, 0.55) 2.01
Dhruva et al. [90] 0.45 (0.43, 0.47) 3.19
Boshara et al. [98] 0.61 (0.42, 0.78) 2.25
Lang et al. [57] 0.64 (0.62, 0.65) 3.21
Singh et al. [100] 0.52 (0.48, 0.56) 3.15
Hernandez-Montfort et al. [101] 0.45 (0.37, 0.54) 2.95
Kondo et al. [115] 0.14 (0.00, 0.58) 1.14
Pieri et al. [45] 0.39 (0.31, 0.48) 2.95
Kajy et al. [91] 0.72 (0.53, 0.87) 2.20
Lemor et al. [92] 0.27 (0.23, 0.31) 3.12
Esposito et al. [83] 0.57 (0.34, 0.77) 2.04
Karami et al. [51] 0.53 (0.43, 0.64) 2.80
Sonu et al. [97] 0.39 (0.35, 0.42) 3.17
O'Neill et al. [85] 0.51 (0.46, 0.55) 3.13
Garan et al. [86] 0.45 (0.27, 0.64) 2.25
Ouweneel et al. [43] 0.58 (0.48, 0.67) 2.87
Rohm et al. [88] 0.45 (0.38, 0.52) 3.02
O'Neill et al. [75] 0.51 (0.46, 0.56) 3.13
Esposito et al. [78] 0.47 (0.24, 0.71) 1.90
Lazkani et al. [70] 0.21 (0.16, 0.26) 3.06
Subtotal (I^2 = 96.50%, p = 0.00) 0.47 (0.41, 0.53) 59.58
Discharge
Karatolios et al. [58] 0.49 (0.38, 0.61) 2.77
Chatzis et al. [58] 0.59 (0.48, 0.70) 2.76
Nelson et al. [103] 0.26 (0.13, 0.44) 2.31
Diakos et al. [104] 0.40 (0.28, 0.53) 2.65
Ba et al. [82] 0.28 (0.21, 0.35) 2.98
Loehn et al. [121] 0.64 (0.52, 0.75) 2.72
Trpkov et al. [95] 0.41 (0.25, 0.59) 2.31
Yourshaw et al. [84] 0.44 (0.24, 0.65) 2.10
Karatolios et al. [40] 0.50 (0.16, 0.84) 1.24
Garan et al. [86] 0.36 (0.11, 0.69) 1.48
Basir et al. [77] 0.24 (0.12, 0.40) 2.42
Pieri et al. [37] 0.21 (0.08, 0.41) 2.18
Basir et al. [71] 0.66 (0.60, 0.71) 3.07
Meraj et al. [72] 0.61 (0.43, 0.77) 2.34
Vase et al. [36] 0.42 (0.15, 0.72) 1.54
Subtotal (I^2 = 87.12%, p = 0.00) 0.44 (0.34, 0.54) 34.86
To explant
O'Neill et al. [75] 0.27 (0.23, 0.31) 3.13
Basir et al. [77] 0.15 (0.06, 0.29) 2.42
Subtotal (I^2 = .%, p = .) 0.26 (0.22, 0.30) 5.55
Heterogeneity between groups: p = 0.000
Overall (I^2 = 95.55%, p = 0.00); 0.44 (0.40, 0.49) 100.00
0 .1 .2 .3 .4 .5 .6 .7 .8 .9 1
%

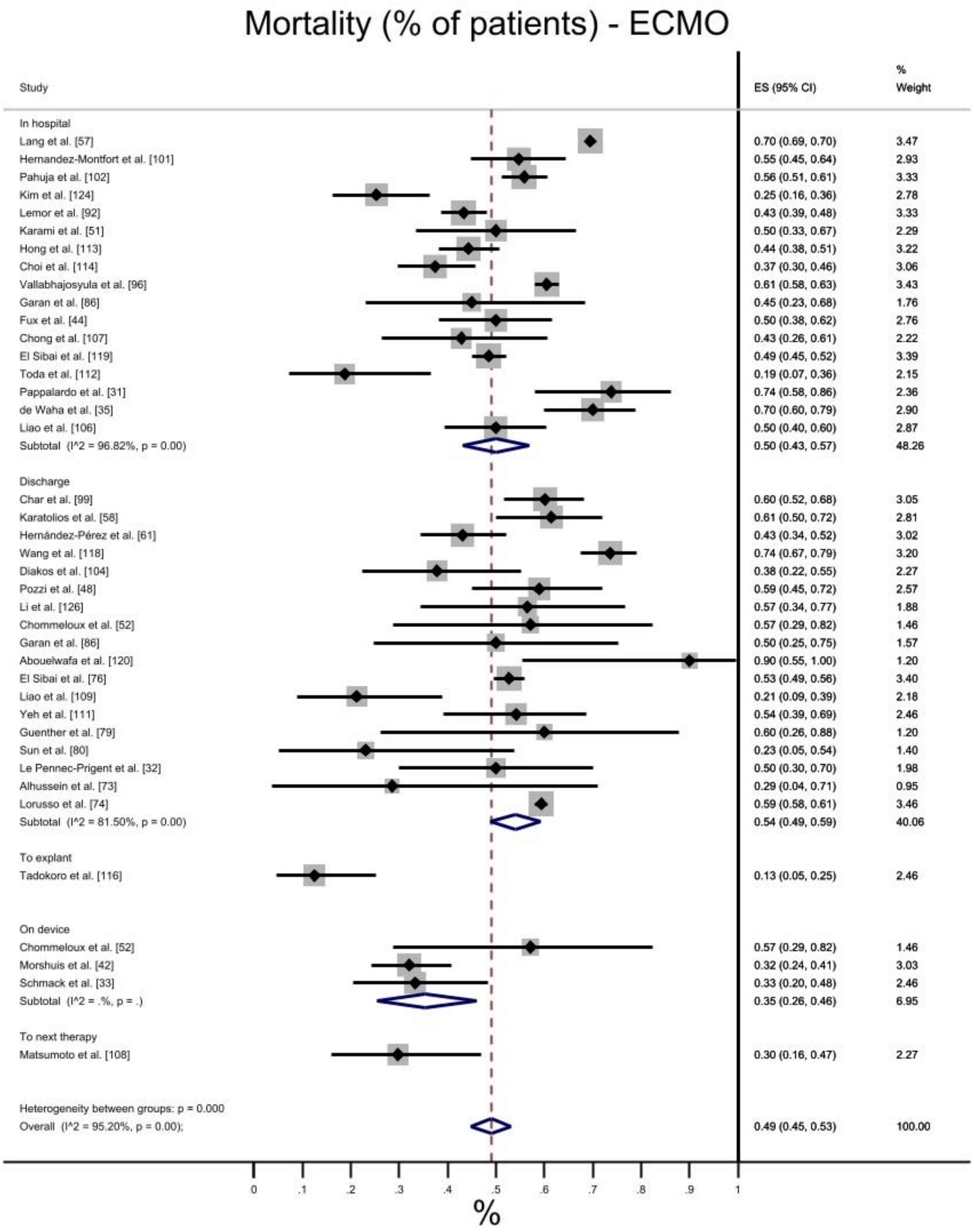
Mortality (% of patients) - ECMO
Study
ES (95% CI)
% Weight
In hospital
Lang et al. [57] 0.70 (0.69, 0.70) 3.47
Hernandez-Montfort et al. [101] 0.55 (0.45, 0.64) 2.93
Pahuja et al. [102] 0.56 (0.51, 0.61) 3.33
Kim et al. [124] 0.25 (0.16, 0.36) 2.78
Lemor et al. [92] 0.43 (0.39, 0.48) 3.33
Karami et al. [51] 0.50 (0.33, 0.67) 2.29
Hong et al. [113] 0.44 (0.38, 0.51) 3.22
Choi et al. [114] 0.37 (0.30, 0.46) 3.06
Vallabhajosyula et al. [96] 0.61 (0.58, 0.63) 3.43
Garan et al. [86] 0.45 (0.23, 0.68) 1.76
Fux et al. [44] 0.50 (0.38, 0.62) 2.76
Chong et al. [107] 0.43 (0.26, 0.61) 2.22
El Sibai et al. [119] 0.49 (0.45, 0.52) 3.39
Toda et al. [112] 0.19 (0.07, 0.36) 2.15
Pappalardo et al. [31] 0.74 (0.58, 0.86) 2.36
de Waha et al. [35] 0.70 (0.60, 0.79) 2.90
Liao et al. [106] 0.50 (0.40, 0.60) 2.87
Subtotal (I^2 = 96.82%, p = 0.00) 0.50 (0.43, 0.57) 48.26
Discharge
Char et al. [99] 0.60 (0.52, 0.68) 3.05
Karatolios et al. [58] 0.61 (0.50, 0.72) 2.81
Hernández-Pérez et al. [61] 0.43 (0.34, 0.52) 3.02
Wang et al. [118] 0.74 (0.67, 0.79) 3.20
Diakos et al. [104] 0.38 (0.22, 0.55) 2.27
Pozzi et al. [48] 0.59 (0.45, 0.72) 2.57
Li et al. [126] 0.57 (0.34, 0.77) 1.88
Chommeloux et al. [52] 0.57 (0.29, 0.82) 1.46
Garan et al. [86] 0.50 (0.25, 0.75) 1.57
Abouelwafa et al. [120] 0.90 (0.55, 1.00) 1.20
El Sibai et al. [76] 0.53 (0.49, 0.56) 3.40
Liao et al. [109] 0.21 (0.09, 0.39) 2.18
Yeh et al. [111] 0.54 (0.39, 0.69) 2.46
Guenther et al. [79] 0.60 (0.26, 0.88) 1.20
Sun et al. [80] 0.23 (0.05, 0.54) 1.40
Le Pennec-Prigent et al. [32] 0.50 (0.30, 0.70) 1.98
Alhussein et al. [73] 0.29 (0.04, 0.71) 0.95
Lorusso et al. [74] 0.59 (0.58, 0.61) 3.46
Subtotal (I^2 = 81.50%, p = 0.00) 0.54 (0.49, 0.59) 40.06
To explant
Tadokoro et al. [116] 0.13 (0.05, 0.25) 2.46
On device
Chommeloux et al. [52] 0.57 (0.29, 0.82) 1.46
Morshuis et al. [42] 0.32 (0.24, 0.41) 3.03
Schmack et al. [33] 0.33 (0.20, 0.48) 2.46
Subtotal (I^2 = .%, p = .) 0.35 (0.26, 0.46) 6.95
To next therapy
Matsumoto et al. [108] 0.30 (0.16, 0.47) 2.27
Heterogeneity between groups: p = 0.000
Overall (I^2 = 95.20%, p = 0.00); 0.49 (0.45, 0.53) 100.00
0 .1 .2 .3 .4 .5 .6 .7 .8 .9 1
%

Figure S2. Bridge to LVAD: Impella and VA-ECMO

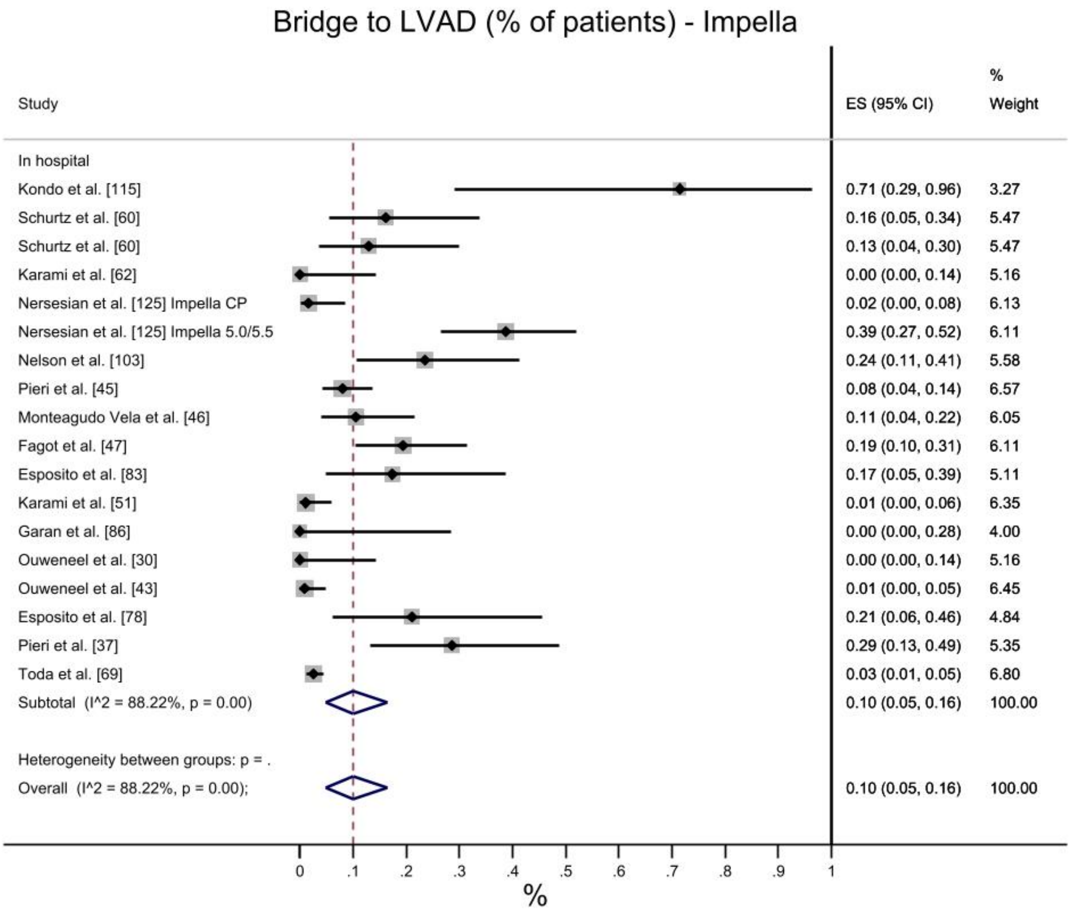

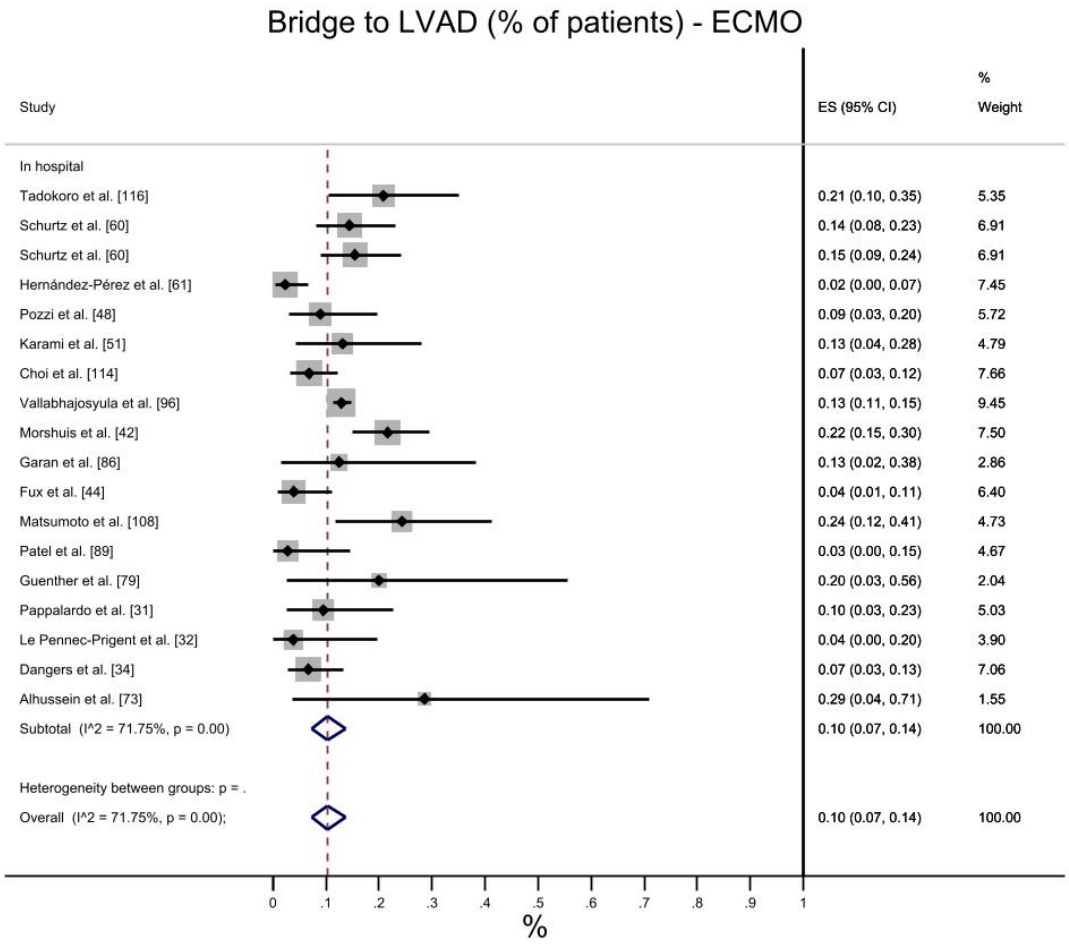
Bridge to LVAD (% of patients) - ECMO
Study
ES (95% CI)
% Weight
In hospital
Tadokoro et al. [116] 0.21 (0.10, 0.35) 5.35
Schurtz et al. [60] 0.14 (0.08, 0.23) 6.91
Schurtz et al. [60] 0.15 (0.09, 0.24) 6.91
Hernández-Pérez et al. [61] 0.02 (0.00, 0.07) 7.45
Pozzi et al. [48] 0.09 (0.03, 0.20) 5.72
Karami et al. [51] 0.13 (0.04, 0.28) 4.79
Choi et al. [114] 0.07 (0.03, 0.12) 7.66
Vallabhajosyula et al. [96] 0.13 (0.11, 0.15) 9.45
Morshuis et al. [42] 0.22 (0.15, 0.30) 7.50
Garan et al. [86] 0.13 (0.02, 0.38) 2.86
Fux et al. [44] 0.04 (0.01, 0.11) 6.40
Matsumoto et al. [108] 0.24 (0.12, 0.41) 4.73
Patel et al. [89] 0.03 (0.00, 0.15) 4.67
Guenther et al. [79] 0.20 (0.03, 0.56) 2.04
Pappalardo et al. [31] 0.10 (0.03, 0.23) 5.03
Le Pennec-Prigent et al. [32] 0.04 (0.00, 0.20) 3.90
Dangers et al. [34] 0.07 (0.03, 0.13) 7.06
Alhussein et al. [73] 0.29 (0.04, 0.71) 1.55
Subtotal (I^2 = 71.75%, p = 0.00) 0.10 (0.07, 0.14) 100.00
Heterogeneity between groups: p = .
Overall (I^2 = 71.75%, p = 0.00); 0.10 (0.07, 0.14) 100.00
0 .1 .2 .3 .4 .5 .6 .7 .8 .9 1
%

Figure S3. Bridge to transplant: Impella and VA-ECMO

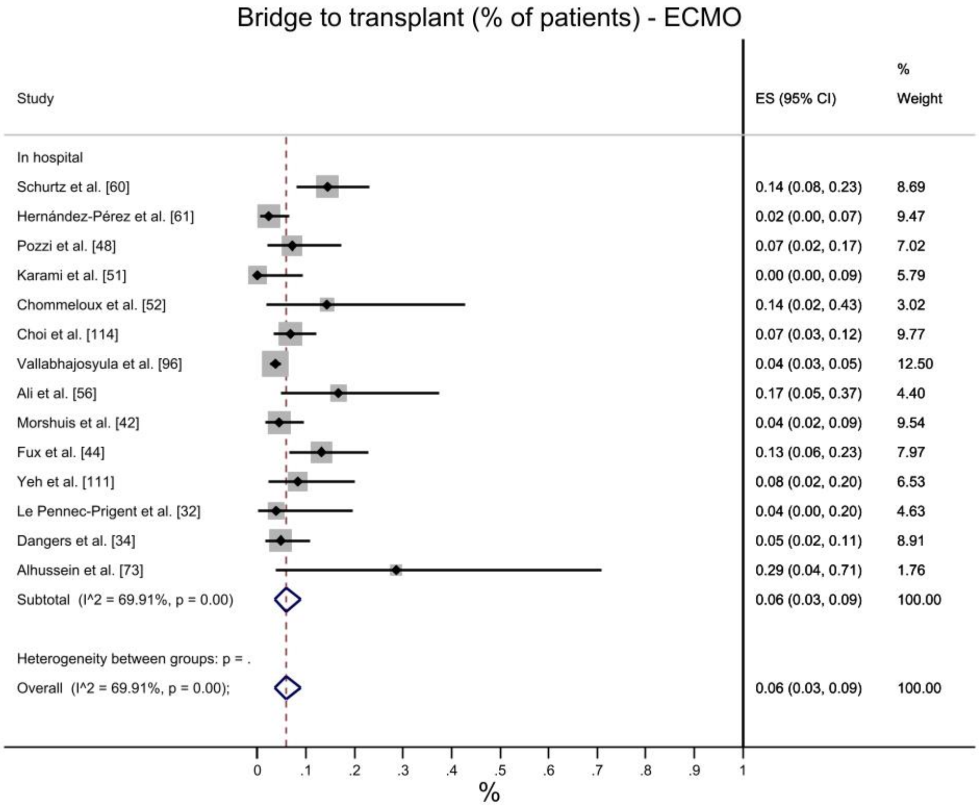

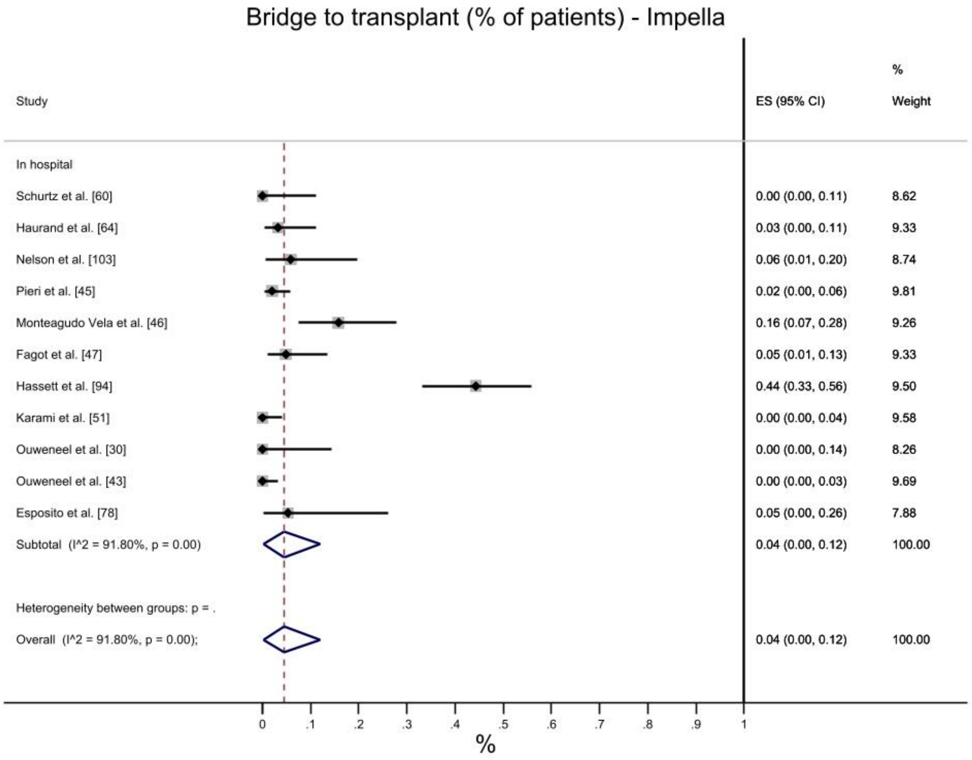

Figure S4. Renal failure: Impella and VA-ECMO

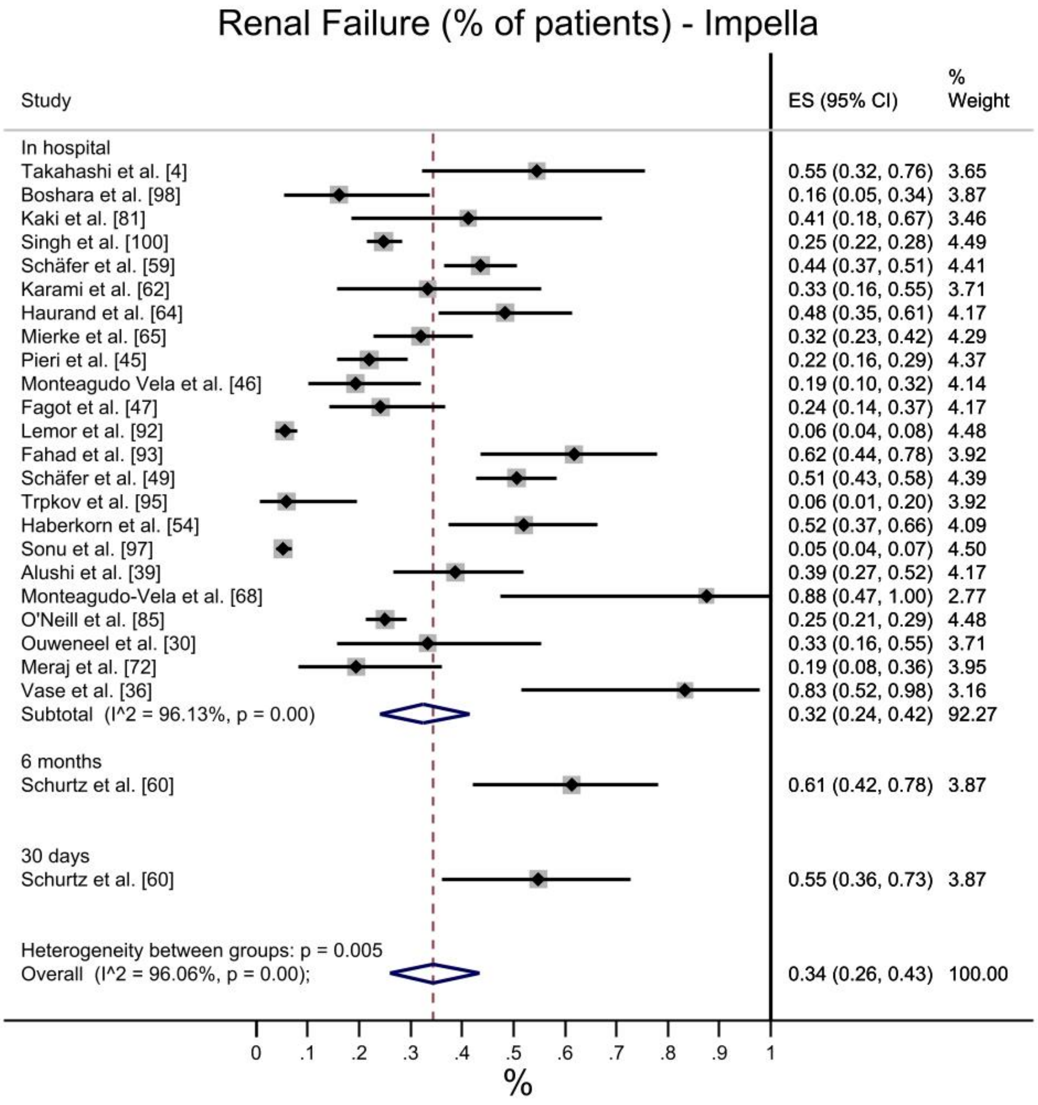

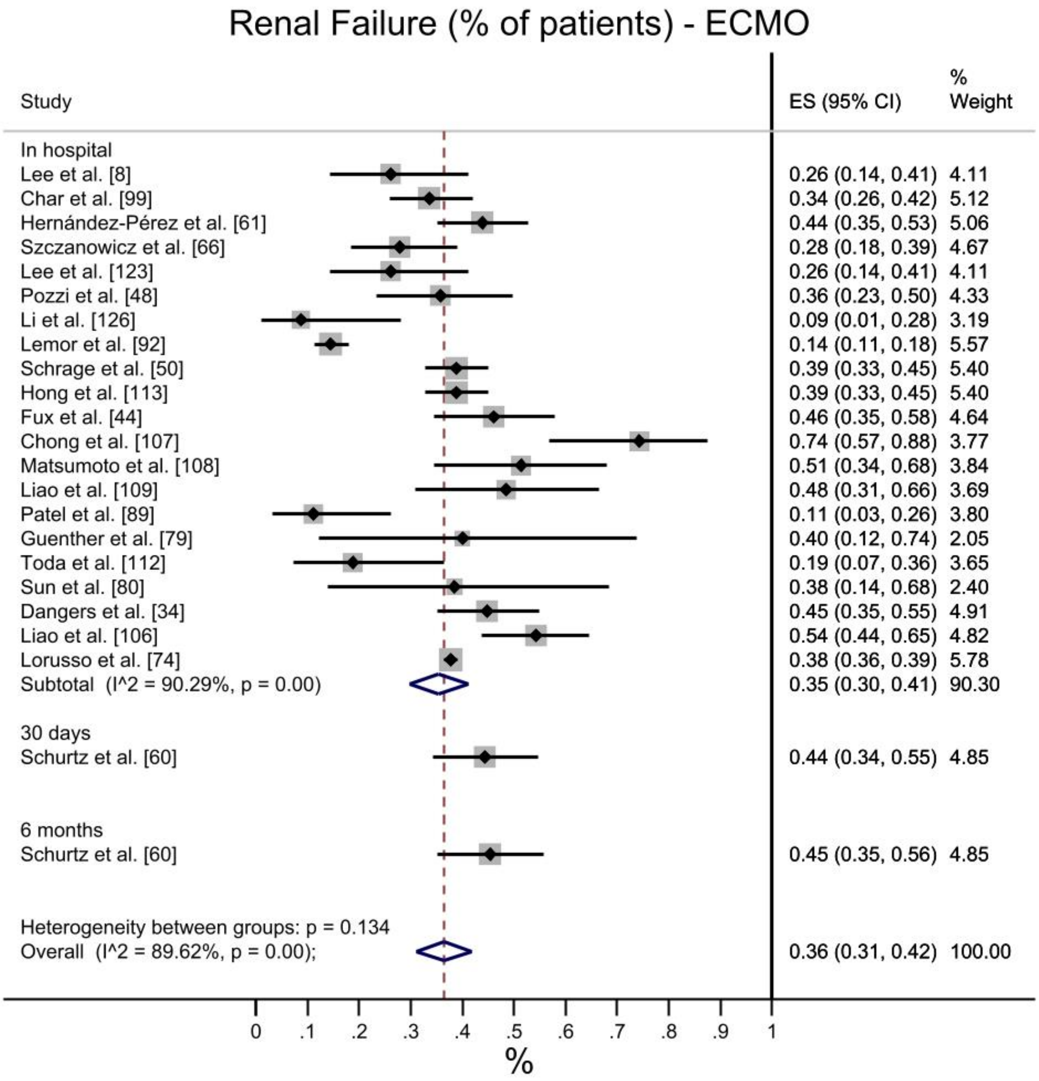
Renal Failure (% of patients) - ECMO
Study
ES (95% CI)
% Weight
In hospital
Lee et al. [8] 0.26 (0.14, 0.41) 4.11
Char et al. [99] 0.34 (0.26, 0.42) 5.12
Hernández-Pérez et al. [61] 0.44 (0.35, 0.53) 5.06
Szczanowicz et al. [66] 0.28 (0.18, 0.39) 4.67
Lee et al. [123] 0.26 (0.14, 0.41) 4.11
Pozzi et al. [48] 0.36 (0.23, 0.50) 4.33
Li et al. [126] 0.09 (0.01, 0.28) 3.19
Lemor et al. [92] 0.14 (0.11, 0.18) 5.57
Schrage et al. [50] 0.39 (0.33, 0.45) 5.40
Hong et al. [113] 0.39 (0.33, 0.45) 5.40
Fux et al. [44] 0.46 (0.35, 0.58) 4.64
Chong et al. [107] 0.74 (0.57, 0.88) 3.77
Matsumoto et al. [108] 0.51 (0.34, 0.68) 3.84
Liao et al. [109] 0.48 (0.31, 0.66) 3.69
Patel et al. [89] 0.11 (0.03, 0.26) 3.80
Guenther et al. [79] 0.40 (0.12, 0.74) 2.05
Toda et al. [112] 0.19 (0.07, 0.36) 3.65
Sun et al. [80] 0.38 (0.14, 0.68) 2.40
Dangers et al. [34] 0.45 (0.35, 0.55) 4.91
Liao et al. [106] 0.54 (0.44, 0.65) 4.82
Lorusso et al. [74] 0.38 (0.36, 0.39) 5.78
Subtotal (I^2 = 90.29%, p = 0.00) 0.35 (0.30, 0.41) 90.30
30 days
Schurtz et al. [60] 0.44 (0.34, 0.55) 4.85
6 months
Schurtz et al. [60] 0.45 (0.35, 0.56) 4.85
Heterogeneity between groups: p = 0.134
Overall (I^2 = 89.62%, p = 0.00); 0.36 (0.31, 0.42) 100.00
0 .1 .2 .3 .4 .5 .6 .7 .8 .9 1
%

Figure S5. Days on support: Impella and VA-ECMO

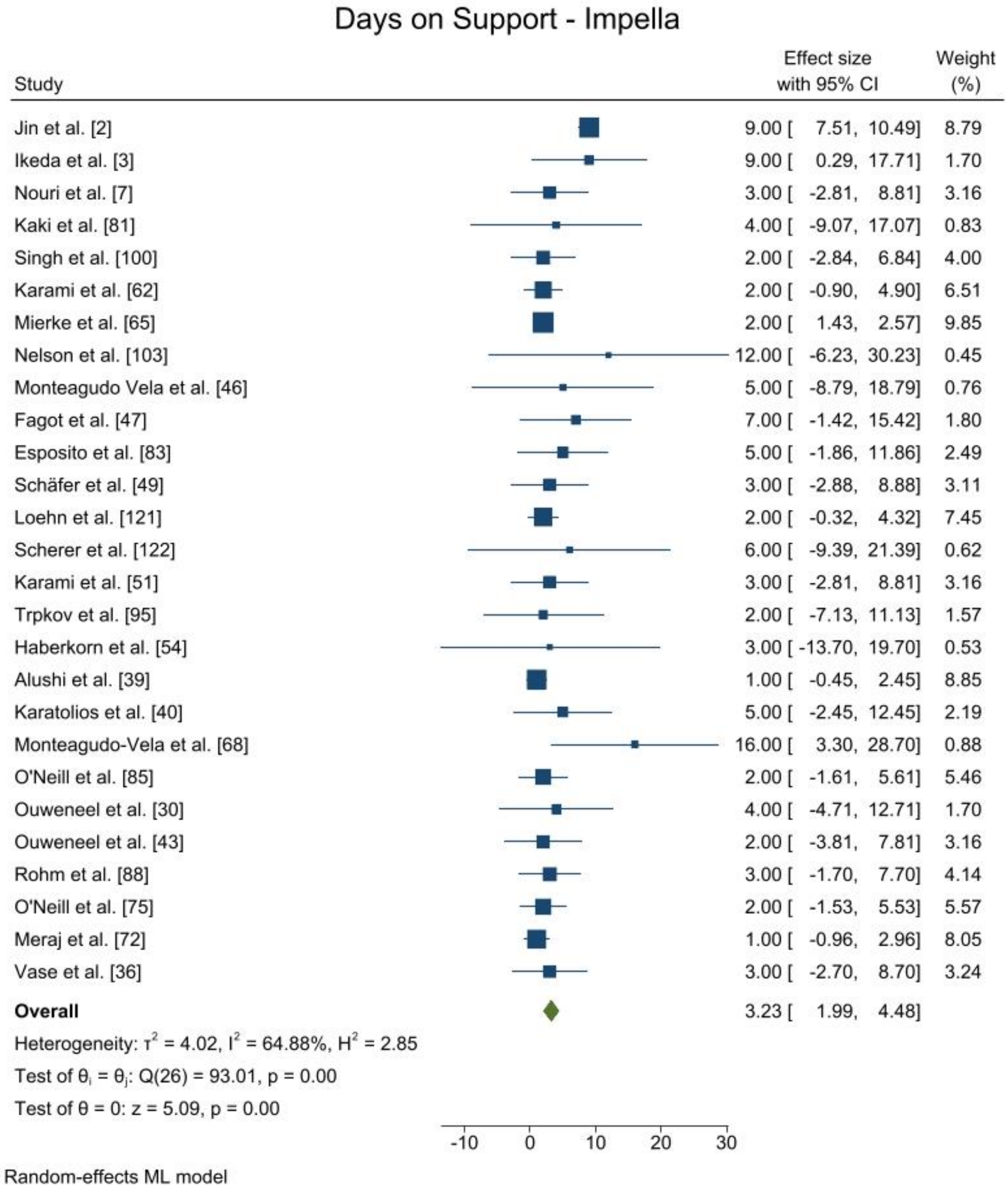

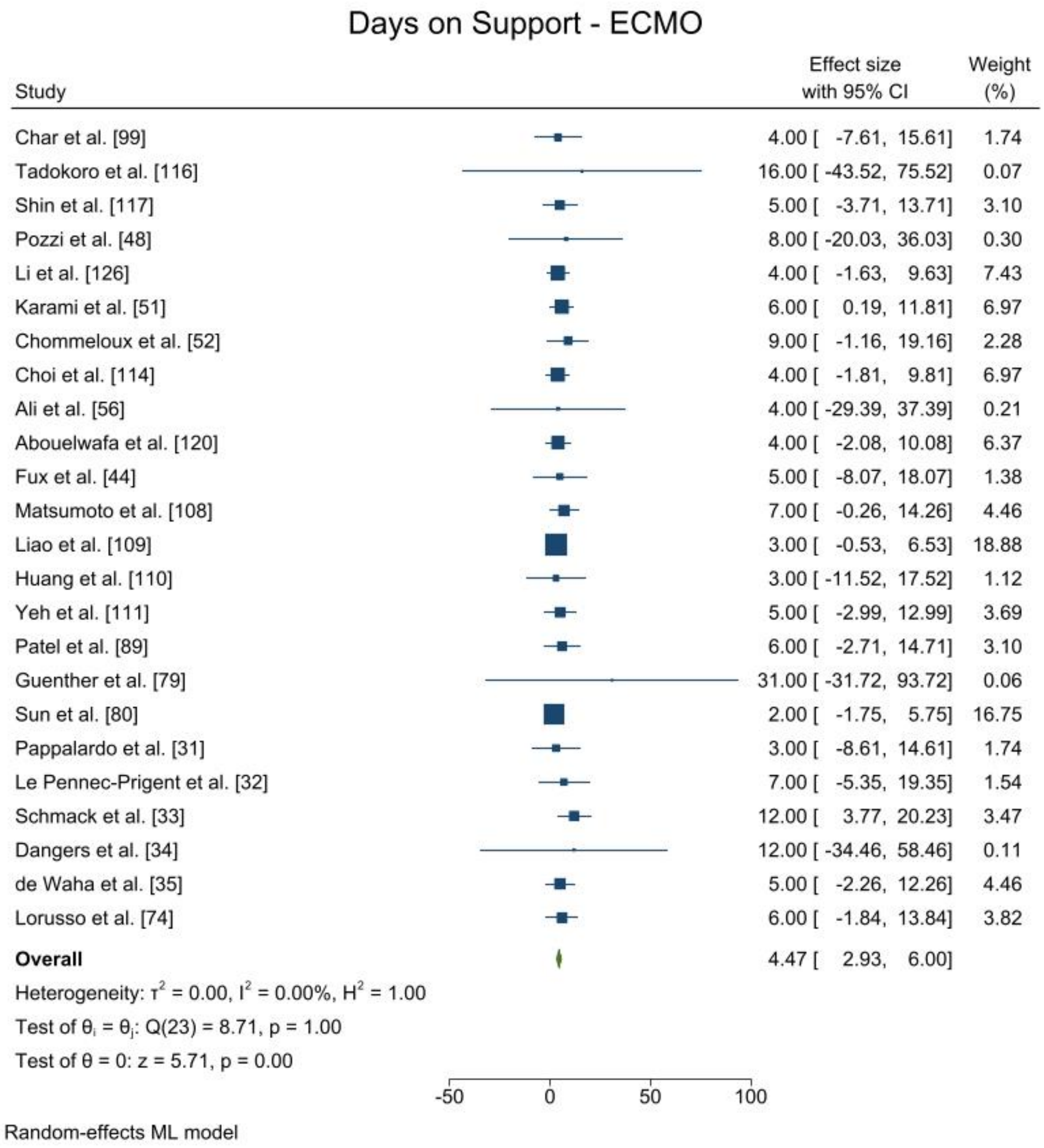
Days on Support - ECMO
Study
Effect size with 95% CI
Weight (%)
Char et al. [99] 4.00 [-7.61, 15.61] 1.74
Tadokoro et al. [116] 16.00 [-43.52, 75.52] 0.07
Shin et al. [117] 5.00 [-3.71, 13.71] 3.10
Pozzi et al. [48] 8.00 [-20.03, 36.03] 0.30
Li et al. [126] 4.00 [-1.63, 9.63] 7.43
Karami et al. [51] 6.00 [0.19, 11.81] 6.97
Chommeloux et al. [52] 9.00 [-1.16, 19.16] 2.28
Choi et al. [114] 4.00 [-1.81, 9.81] 6.97
Ali et al. [56] 4.00 [-29.39, 37.39] 0.21
Abouelwafa et al. [120] 4.00 [-2.08, 10.08] 6.37
Fux et al. [44] 5.00 [-8.07, 18.07] 1.38
Matsumoto et al. [108] 7.00 [-0.26, 14.26] 4.46
Liao et al. [109] 3.00 [-0.53, 6.53] 18.88
Huang et al. [110] 3.00 [-11.52, 17.52] 1.12
Yeh et al. [111] 5.00 [-2.99, 12.99] 3.69
Patel et al. [89] 6.00 [-2.71, 14.71] 3.10
Guenther et al. [79] 31.00 [-31.72, 93.72] 0.06
Sun et al. [80] 2.00 [-1.75, 5.75] 16.75
Pappalardo et al. [31] 3.00 [-8.61, 14.61] 1.74
Le Pennec-Prigent et al. [32] 7.00 [-5.35, 19.35] 1.54
Schmack et al. [33] 12.00 [3.77, 20.23] 3.47
Dangers et al. [34] 12.00 [-34.46, 58.46] 0.11
de Waha et al. [35] 5.00 [-2.26, 12.26] 4.46
Lorusso et al. [74] 6.00 [-1.84, 13.84] 3.82
Overall 4.47 [2.93, 6.00]
Heterogeneity: $\tau^2 = 0.00$, $I^2 = 0.00\%$, $H^2 = 1.00$
Test of $\theta_i = \theta_j$: Q(23) = 8.71, p = 1.00
Test of $\theta = 0$: z = 5.71, p = 0.00
-50 0 50 100
Random-effects ML model

Supplementary Figure S6. Pre-PCI vs. Post-PCI mortality at 30 days

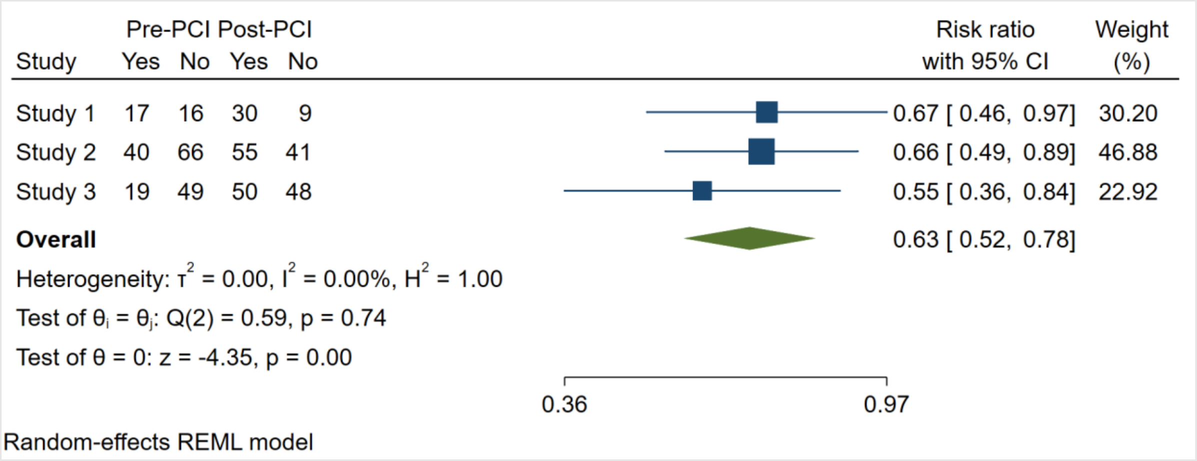

Study 1 = Lohen et al. [121]; Study 2 = Schäfer et al. [49]; Study 3 = Schäfer et al. [122]

Yes = Mortality; No = Survival

Supplementary Figure S7. Pre-PCI vs. Post-PCI Mortality at discharge

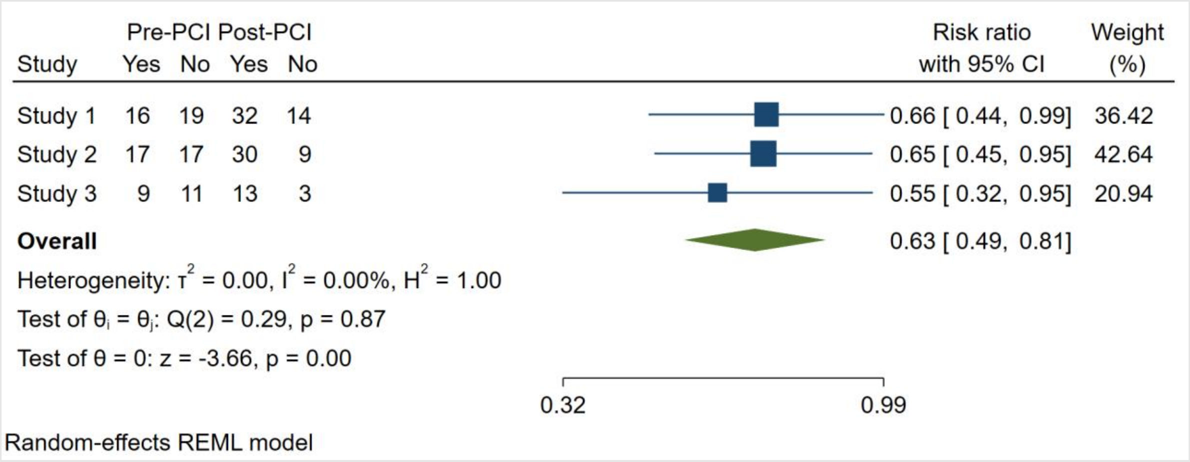

Study 1 = Chatzis et al. [58]; Study 2 = Lohen et al. [121]; Study 3 = Meraj et al. [72]

Yes = Mortality; No = Survival
